# New systematic assignments in Gonyleptoidea (Arachnida, Opiliones, Laniatores)

**DOI:** 10.3897/zookeys.198.2337

**Published:** 2012-05-30

**Authors:** Ricardo Pinto-da-Rocha, Alípio Rezende Benedetti, Eduardo Gomes de Vasconcelos, Marcos Ryotaro Hara

**Affiliations:** 1 Departamento de Zoologia, Instituto de Biociências, Universidade de São Paulo, Caixa Postal 11461, São Paulo, SP, Brazil, 05422-970; 2 Núcleo de Estudos da Biodiversidade da Amazônia Matogrossense, Instituto de Ciências Naturais, Humanas e Sociais, Universidade Federal de Mato Grosso, Av. Alexandre Ferronato, 1200, 78557-267 Sinop, MT, Brazil; 3 Escola de Artes, Ciências e Humanidades, Universidade de São Paulo, Av. Arlindo Bettio no 1000, Ermelino Matarazzo, São Paulo, SP, Brazil , 03828-000

**Keywords:** Agoristenidae, Cranaidae, Gonyleptidae, Neotropics, taxonomy

## Abstract

As part of an ongoing revision of the family Gonyleptidae, we have identified many species that are synonyms of previously described species or misplaced in this family. This article summarizes these findings, adding previously unavailable information or correcting imprecise observations to justify the presented taxonomic changes.

The following new familial or subfamilial assignments are proposed: *Nemastygnus* Roewer, 1929 and *Taulisa* Roewer, 1956 are transferred to Agoristenidae, Agoristeninae; *Napostygnus* Roewer, 1929 to Cranaidae; *Ceropachylinus peruvianus* Roewer, 1956 and *Pirunipygus* Roewer, 1936 are transferred to Gonyleptidae, Ampycinae; *Gyndesops* Roewer, 1943, *Haversia* Roewer, 1913 and *Oxapampeus* Roewer, 1963 are transferred to Gonyleptidae, Pachylinae.

The following generic synonymies are proposed for the family Gonyleptidae: *Acanthogonyleptes* Mello-Leitão, 1922 = *Centroleptes* Roewer, 1943; *Acrographinotus* Roewer, 1929 = *Unduavius* Roewer, 1929; *Gonyleptes* Kirby, 1819 = *Collonychium* Bertkau, 1880; *Mischonyx* Bertkau, 1880 = *Eugonyleptes* Roewer, 1913 and *Gonazula* Roewer, 1930; *Parampheres* Roewer, 1913 = *Metapachyloides* Roewer, 1917; *Pseudopucrolia* Roewer, 1912 = *Meteusarcus* Roewer, 1913; *Haversia* Roewer, 1913 = *Hoggellula* Roewer, 1930.

The following specific synonymies are proposed for the family Gonyleptidae: *Acanthogonyleptes singularis* (Mello-Leitão, 1935) = *Centroleptes flavus* Roewer, 1943, **syn. n.**; *Geraeocormobius sylvarum* Holmberg, 1887 = *Discocyrtus serrifemur* Roewer, 1943, **syn**. **n.**; *Gonyleptellus bimaculatus* (Sørensen, 1884) = *Gonyleptes cancellatus* Roewer,1917, **syn. n.**; *Gonyleptes atrus* Mello-Leitão, 1923 = *Weyhia brieni* Giltay, 1928, **syn. n.**; *Gonyleptes fragilis* Mello-Leitão, 1923 = *Gonyleptes banana* Kury, 2003, **syn. n.**; *Gonyleptes horridus* Kirby, 1819 = *Collonychium bicuspidatum* Bertkau, 1880, **syn. n.**, *Gonyleptes borgmeyeri* Mello-Leitão, 1932, **syn. n.**, *Gonyleptes curvicornis* Mello-Leitão, 1932, **syn. n.**, *Metagonyleptes hamatus* Roewer, 1913, **syn. n.** and *Paragonyleptes simoni* Roewer, 1930, **syn. n.**; *Gonyleptes pustulatus* Sørensen, 1884 = *Gonyleptes guttatus* Roewer, 1917, **syn. n.**; *Haversia defensa* (Butler, 1876) = *Sadocus vallentini* Hogg, 1913, **syn. n.**; *Liogonyleptoides minensis* (Piza, 1946) = *Currala bahiensis* Soares, 1972, **syn. n.**; *Megapachylus grandis* Roewer, 1913 = *Metapachyloides almeidai* Soares & Soares, 1946, **syn. n.**; *Mischonyx cuspidatus* (Roewer, 1913) = *Gonazula gibbosa* Roewer, 1930 **syn. n.**; *Mischonyx scaber* (Kirby, 1819) = *Xundarava holacantha* Mello-Leitão, 1927, **syn. n.**; *Parampheres tibialis* Roewer, 1917 = *Metapachyloides rugosus* Roewer, 1917, **syn. n.**; *Parapachyloides uncinatus* (Sørensen, 1879) = *Goyazella armata* Mello-Leitão, 1931, **syn. n.**; *Pseudopucrolia mutica* (Perty, 1833) = *Meteusarcus armatus* Roewer, 1913, **syn. n.**

The following new combinations are proposed: *Acrographinotus ornatus* (Roewer, 1929), **comb. n.** (ex *Unduavius*); *Gonyleptellus bimaculatus* (Sørensen, 1884),**comb. n.** (ex *Gonyleptes*);*Gonyleptes perlatus* (Mello-Leitão, 1935), **comb. n.** (ex*Moojenia*);*Mischonyx scaber* (Kirby, 1819), **comb. n.** (ex *Gonyleptes*); and *Neopachyloides peruvianus* (Roewer, 1956), **comb. n.** (ex *Ceropachylus*).

The following species of Gonyleptidae, Gonyleptinae are revalidated: *Gonyleptes atrus* Mello-Leitão, 1923 and *Gonyleptes curvicornis* (Roewer, 1913).

## Introduction

Opiliones is currently divided into four monophyletic suborders (Gonzalo and Kury 2007), of which Laniatores, with 29 families and 4040 described species (Kury 2011), is the most diverse. Indeed, it is the main component of tropical opilionid faunas. Recently the Neotropical harvestmen (mainly Gonyleptidae and Cranaidae) have been the subject of many revisions (e.g., DaSilva and Pinto-da-Rocha 2010; Bragagnolo and Pinto-da-Rocha 2009; Yamaguti and Pinto-da-Rocha 2009; Pinto-da-Rocha and Villarreal-Manzanilla 2009; Orrico and Kury 2009), and their subfamilial relationships have gradually been made clear (for instance, compare the above mentioned articles with Kury 1994a). These efforts aim to reverse the twentieth-century classification system in harvestmen systematics known as the “Roewerian system,” which was based on few characteristics and emphasized morphological differences. In addition, species sampling was poor, and intraspecific variation was overlooked. Thus many monotypic and/or artificial groups were created in this period, especially for Neotropical harvestmen. In order to resolve the taxonomically confusing Laniatores, modern studies are based on the examination of the type material as well as other material deposited in museum collections to determine the species identity. This part is especially important because the former classification system did not take intraspecific variations into account. Another important step is to gather overlooked information, such as those from male genitalia, which have shown to be quite reliable as indicative of monophyletic groups (e.g., Pinto-da-Rocha and Hara 2009; Kury and Pinto-da-Rocha 2007a).

From recent phylogenetic studies on the Gonyleptidae subfamilies Gonyleptinae, Pachylinae, and Metasarcinae, as well as visits to such European museums as the Senckenberg Museum (Frankfurt), Muséum National d’Histoire Naturelle (Paris), and Zoologisches Institut and Zoologisches Museum (Hamburg), we were able to precisely identify many gonyleptids, resulting in the detection of numerous synonymies and the misplacement of several species relative to modern family and subfamily concepts. Thus, we decided to publish these systematic findings in this format instead of many revisions of the respective families/subfamilies, which would take several years to finish. The aim of this article is to propose nomenclatural changes in an attempt to resolve the taxonomic nightmare in Neotropical harvestmen systematics.

## Material and methods

The following abbreviations were adopted (curators in parentheses) to refer to the depositories:

NHMThe Natural History Museum [formerly British Museum], London, England (Janet Beccaloni).

HEMSHélia Eller Monteiro Soares private collection, now included in MNRJ.

IBSPInstituto Butantan, São Paulo, São Paulo, Brazil (Darci M. Barros Battesti).

ISNBInstitut Royal des Sciences Naturelles de Belgique, Brussels, Belgium (Léon Baert).

MNRJ Museu Nacional do Rio de Janeiro, Rio de Janeiro, Rio de Janeiro, Brazil (Adriano Brilhante Kury).

MZLQMuseu de Zoologia Luiz de Queiroz, now housed in IBSP.

MZSPMuseu de Zoologia da Universidade de São Paulo, São Paulo, São Paulo, Brazil (Ricardo Pinto-da-Rocha).

NHMWNaturhistorisches Museum, Vienna, Austria (Jürgen Gruber).

SMFNaturmuseum Senckenberg, Frankfurt am Main, Germany (Peter Jäger).

ZMBInstitut für Systematische Zoologie, Museum für Naturkunde der Humboldt-Universität zu Berlin, Germany (Jason Dunlop).

ZMHZoologisches Museum Hamburg, Germany (Hieronymus Dastich).

ZMUCZoologisk Museum, Universität København, Zoological Museum, University of Copenhagen, Denmark (Nikolaj Scharff).

We did not exhaustively cite literature in synonymic listings (for this purpose see Kury 2003), but we updated it when necessary. We also placed information regarding the type material altogether. The list of material examined is only cited if more than one vial other than the type material was examined. Pictures of specimens were taken using a Canon EOS digital camera and edited using Adobe Photoshop and Corel PhotoPaint computer software. The illustrations of the external morphology were made under LEICA MZAPO stereomicroscope using camera lucida. Male genitalia were prepared according to Pinto-da-Rocha (1997) for illustrations as well as SEM (Scanning Electronic Microscope) pictures. In the redescriptions, nomenclature of structures and relative positions follow Acosta et al. (2007b), with some modifications to best fit the taxa. Prolateral and retrolateral setae formulae of pedipalpal tibia and tarsi follow Pinto-da-Rocha (1997). The prosomal part of dorsal scutum and the scutal area V are here called the “carapace” and “posterior margin of dorsal scutum”, respectively. Measurements of the body parts (except for genitalia) are in millimeters.

## Taxonomy

### Agoristenidae Šilhavý, 1973

Leiosteninae Šilhavý, 1973

#### Nemastygnus

Roewer, 1929 new family assignment

http://species-id.net/wiki/Nemastygnus

Nemastygnus Roewer, 1929: 277; Kury 2003: 145; (type species: *Nemastygnus ovalis* Roewer, 1929, by monotypy).

##### Diagnosis.

*Nemastygnus* was transferred from Cranaidae to Gonyleptidae, Metasarcinae by Kury (2003). It is closely related to *Avima* Roewer, 1949, based on dorsal scutum shape (rectangular) and scutal area III (unarmed). It is impossible to distinguish both genera mainly because *Avima*, the largest genus of Leiosteninae with 33 species, is a heterogeneous genus based on penial characters. The type-species of *Avima*, *Avima leucobunus* Roewer, 1949, is known only from the original description (and only external morphology). However, we will not propose synonymy in this paper because *Nemastygnus* is older and 33 new combinations should be proposed. A review of *Avima* plus *Nemastygnus* species is needed.

##### Description.

**Figure 1.**
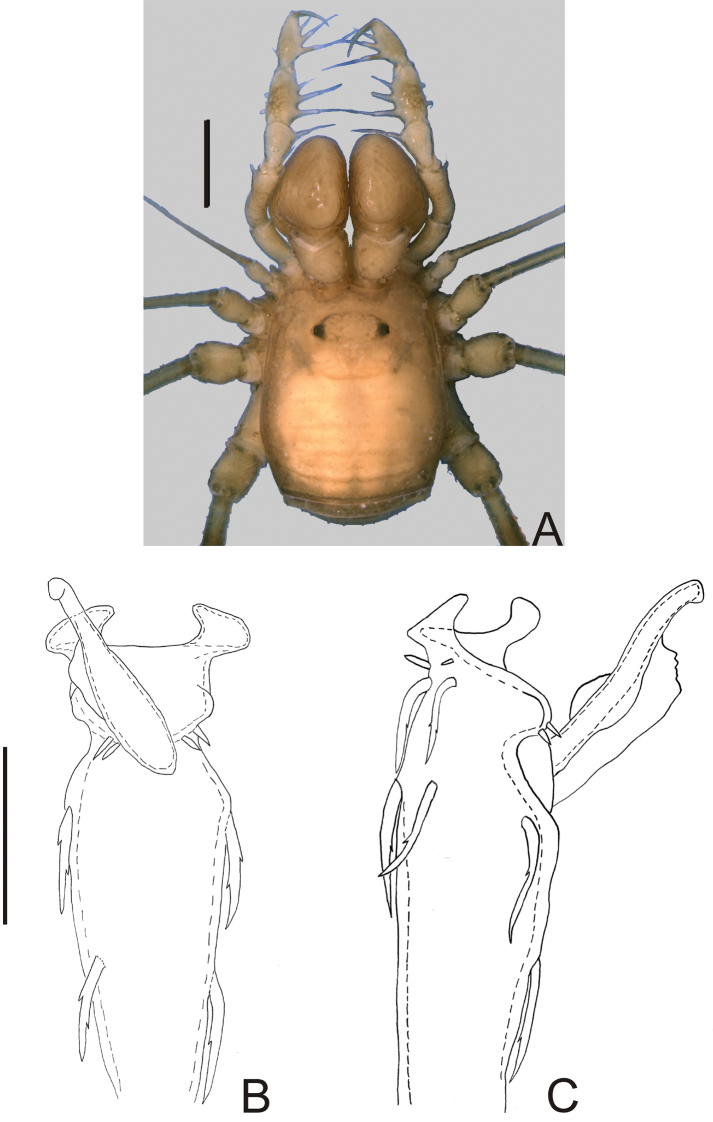
*Nemastygnus ovalis* Roewer. Male (holotype): **A** habitus, dorsal view. Distal part of penis **B** in dorsal view **C** ditto, left lateral view. **B, C** at same scale. Sale bars: **A** = 1 mm; **B** = 0.1 mm.

Ocularium unarmed, saddle-shaped. Areas and posterior margin of dorsal scutum and free tergites unarmed (Fig. 1A). Scutal area I undivided. Lateral margin of dorsal scutum coriaceus. Chelicera dimorphic. Pedipalp with slender articles; femur without dorsoapical spine, with row of three ventral large setae and two ventroapical large setae; patella with prolateral large setae; tibia–tarsus with ectal-mesal large setae. Leg I filiform, three-segmented. Penis: basal setae of truncus and basal setae of ventral plate very long and bifid; stylus with a large, longitudinal keel.

#### Nemastygnus ovalis

Roewer, 1929

http://species-id.net/wiki/Nemastygnus_ovalis

Fig. 1

Nemastygnus ovalis Roewer, 1929: 277, fig. 44 (♂); Kury 2003: 145; (male holotype, Colombia, Cundinamarca, Bogotá, SMF RI 1005/4, examined).

##### Description.

Penis (Fig. 1B–C; holotype): truncus with three pairs of bifid setae (basal, subdistal lateral and distal ventral). Ventral plate with rounded lobe on corners, two dorso-basal pairs of small single branched setae, a ventral distal pair of small single branched setae and a ventral median bifid setae. Glans dorsally projected, slender, with a small ventral crest and a long dorsal crest. Stylus smooth.

##### Taxonomical note.

*Nemastygnus* was originally placed in Gonyleptidae, Prostygninae. Kury (1994b) transferred Prostygninae to Cranaidae. Later, in his catalogue, Kury (2003) transferred *Nemastygnus* to Gonyleptidae, Metasarcinae without examining the type material. We herein propose the transfer of *Nemastygnus* to Agoristenidae Leiosteninae, based on characteristics of body and male genitalia, viz., filiform leg I, saddle-shaped ocularium, pedipalpus with well developed setae in ventral row of femur (basalmost longest, size including socket about length of pedipalpal femur) and prolateral/retrolateral of tibia and tarsus, penis with typical ventral plate and bifid setae (Kury 1993; Pinto-da-Rocha 1996; Kury 1997; Pinto-da-Rocha and Hara 2009).

#### Taulisa

Roewer, 1956 new family assignment

http://species-id.net/wiki/Taulisa

Taulisa Roewer, 1956: 433; Kury 2003: 145; (type species: *Taulisa koepckei* Roewer, 1956, by original designation).

##### Diagnosis.

**Figure 2.**
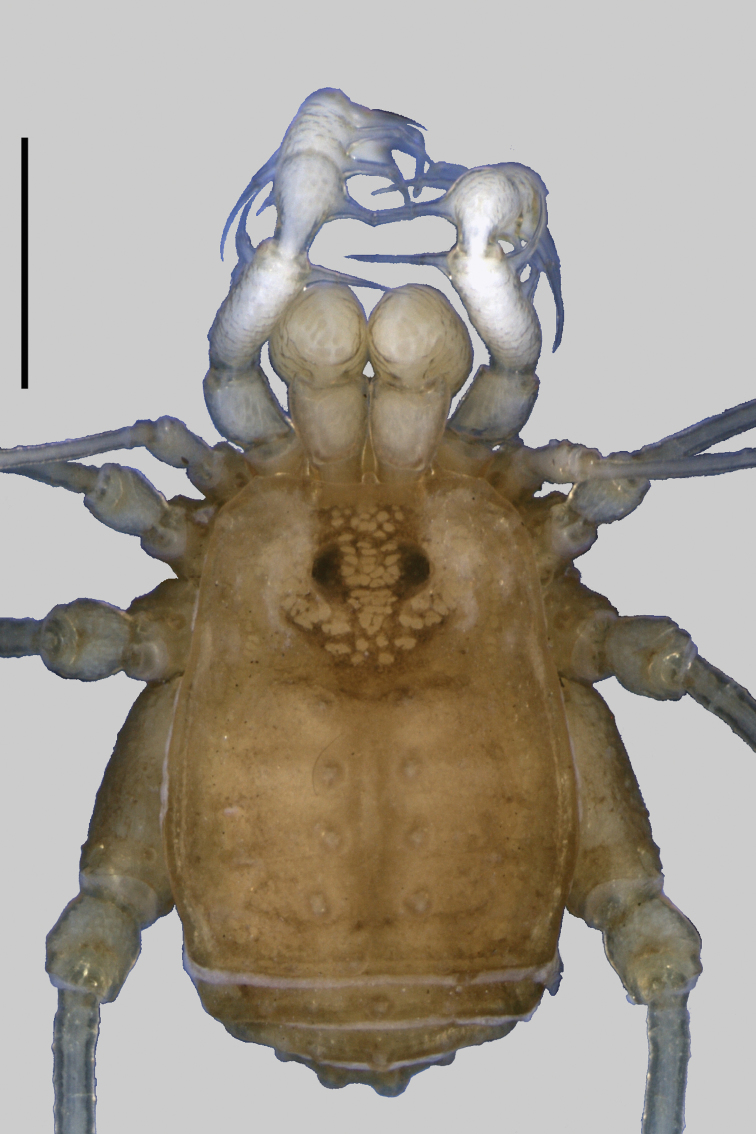
*Taulisa koepckei* Roewer. Female (holotype): Habitus, dorsal view. Scale bar: 1 mm.

*Taulisa* (Fig. 2) differs from the other 10 genera of Leiosteninae by the following combination of characters: ocularium unarmed, saddle-shaped; areas of dorsal scutum with two large tubercles each, posterior margin of dorsal scutum and free tergites, each one with a central large tubercle; area I undivided; lateral margin of dorsal scutum coriaceus; pedipalp with slender articles, its femur with dorsoapical spine, with row of four ventral large setae; pedipalpal patella with prolateral large setae; pedipalpal tibia–tarsus with ectal-mesal large setae; leg I filiform, three-segmented. Male unknown.

##### Taxonomical note.

*Taulisa* was originally placed in Phalangodidae, Tricommatinae. Kury (1992a) elevated Tricommatinae to family level. In 2003, Kury transferred *Taulisa* from Tricommatinae to Gonyleptidae, Metasarcinae without further remarks. We propose to transfer *Taulisa* to Agoristenidae, Leiosteninae based on characteristics of the body, viz., filiform leg I, saddle-shaped ocularium, pedipalpus with well developed and long setae in ventral row of femur and on prolateral/retrolateral of tibiae/tarsi (Kury 1993; Pinto-da-Rocha 1996; Kury 1997; Pinto-da-Rocha and Hara 2009). Information regarding male genitalia was unavailable, since this species is only known from the female holotype.

##### Composition.

Monotypic.

#### Taulisa koepckei

Roewer, 1956

http://species-id.net/wiki/Taulisa_koepckei

Fig. 2

Taulisa koepckei Roewer, 1956: 433, fig. 3–4 (♀); Kury 2003: 145; (female holotype, Peru, Lambayeque, Hacienda Taulis, 6°50'S, 79°10'W, 1700 m, SMF 9697, examined).

##### Diagnosis.

As for genus.

### Cranaidae Roewer, 1913

#### Napostygnus

Roewer, 1929 new family assignment

http://species-id.net/wiki/Napostygnus

Napostygnus Roewer, 1929: 274, 275; Kury 2003: 145; (type species: *Napostygnus bispinosus* Roewer, 1929, by monotypy).

##### Diagnosis.

*Napostygnus* was originally placed in Gonyleptidae, Prostygninae and transferred to the Gonyleptidae, Metasarcinae by Kury (2003). It differs from the other 75 genera of Cranaidae by the following combination of characters: ocularium, scutal areas I–IV, posterior margin of dorsal scutum and free tergite I unarmed; free tergites II–III with one spine; posterior margin of dorsal scutum concave; legs thin and unarmed; penis with ventral plate wider at the middle, three pairs of distal setae, two pairs of basal setae, glans with dorsal process, stylus with apex enlarged.

##### Composition.

Monotypic.

#### Napostygnus bispinosus

Roewer, 1929

http://species-id.net/wiki/Napostygnus_bispinosus

Fig. 3

Napostygnus bispinosus Roewer, 1929: 275, fig. 42 (♀); Kury 2003: 145; (female holotype, Ecuador, Napo, Valley of Rio Napo, SMF RI 1004/3, examined).

##### Material examined.

ECUADOR. Napo: Valley of Rio Napo, 1 female holotype (SMF RI 1004/3); Cantón Quijos, Parroquira Cozanga, Yanayacu Research Station (0°35'S, 78°57'W, 2128 m), 1 ♂ & 2 ♀ (MZSP 36132); ditto, 1 ♀ (IBSP 10550).

##### Description.

**Figure 3.**
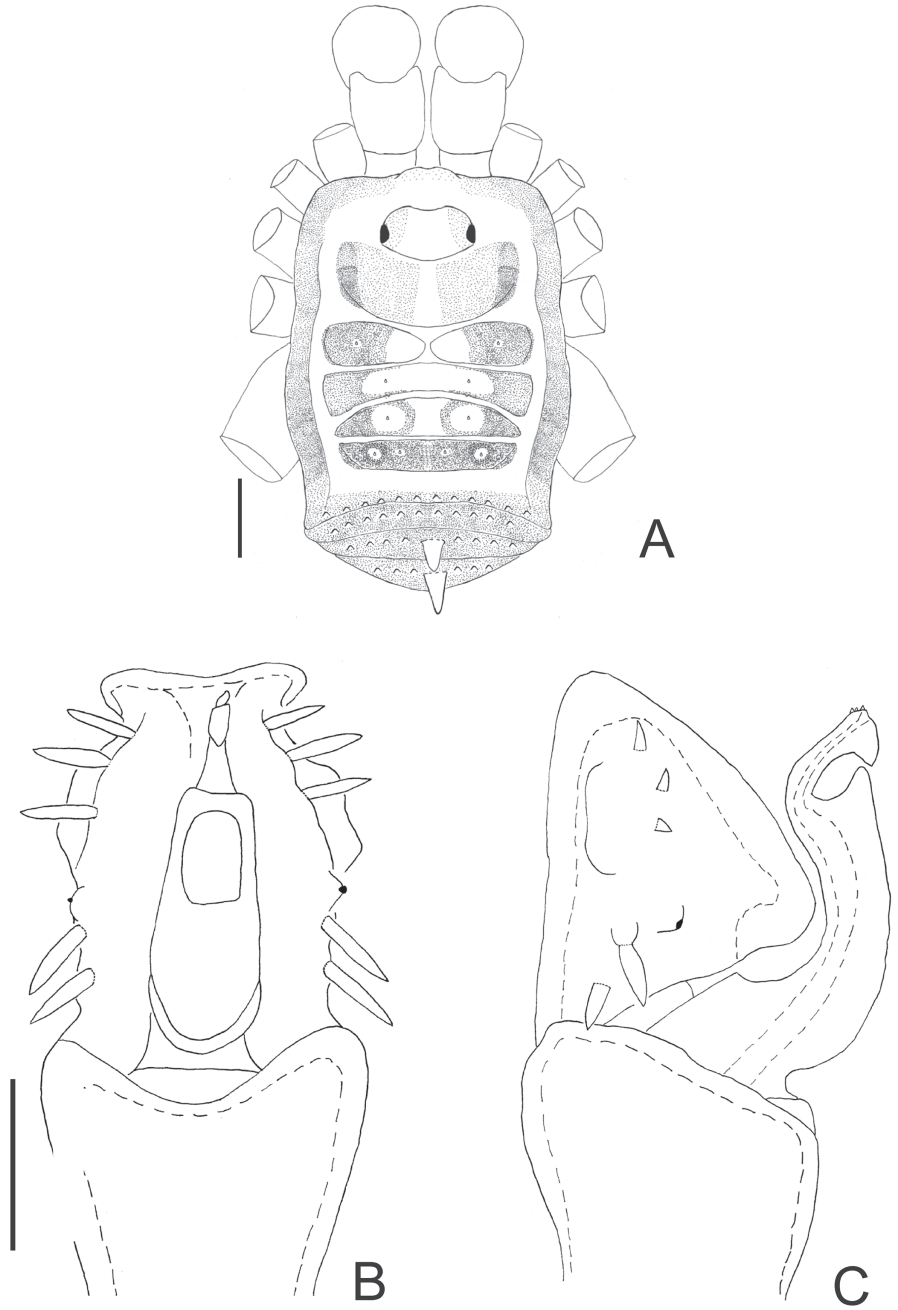
*Napostygnus bispinosus* Roewer. Male (MZSP 36132): **A** habitus, dorsal view. Distal part of penis **B** in dorsal view **C** ditto, left lateral view. **B, C** at same scale. Scale bar **A** = 1 mm. Scale bar **B** = 0.1 mm.

*Male* (MZSP 36132). Dorsum (Fig. 3A). Measurements: dorsal scutum length 4.1; dorsal scutum maximum width 3.5; carapace length 2.1; carapace maximum width 2.9; femur IV length 11.2. Body outline nearly subrectangular. Anterior margin of dorsal scutum with a median frontal hump small-granulate. Ocularium near middle of carapace, saddle shaped, with small granules near the eyes, unarmed. Carapace higher than the rest of dorsal scutum, with 4 tubercles behind ocularium. Scutal areas I–III with 2 small median tubercles on each area; IV with 4 tubercles. Lateral margin of dorsal scutum with a low density of small granules. Posterior margin of dorsal scutum and free tergite I with a row of small granules, unarmed. Free tergites II–III each with a median spine, small granulate.

Chelicera: segment I unarmed. Segment II swollen, finger II with 3 teeth, III with 4 teeth.

Pedipalpus: trochanter with 2 ventral tubercles, femur and patella smooth. Tibial setation: retrolateral iiIii; prolateral iiiIiii. Tarsal setation: retrolateral IiIi; prolateral IiiiIi.

Legs: legs I–IV unarmed and without granules, except for trochanters, which are small granulate. Basitarsus I slightly inflated. Tarsal process present. Tarsal segmentation: 6(3); 16(3); 6; 7.

Penis (Fig. 3B–C): ventral plate with almost straight distal margin, thick median lobe and folded ventrally to the distal setae, 3 pairs of distal setae and 2 pairs of basal setae. Glans with thumb-like dorsal process. Stylus with dorsal apical projection and ventral apical small trichomes.

Coloration (in ethanol) (Fig. 3A): body background yellow with brown spots mainly on carapace, scutal areas, lateral and posterior margins of dorsal scutum and free tergites. Mesotergum with one longitudinal yellow stripe surrounded by blackish pigment at grooves I–V. Pedipalpus and chelicera yellowish brown with a brown reticulate pattern. Legs yellowish brown.

##### Taxonomical note.

*Napostygnus* was originally placed in Gonyleptidae, Prostygninae. Kury (1994b) transferred Prostygninae to Cranaidae and later transferred *Napostygnus* to Gonyleptidae, Metasarcinae (Kury 2003). We herein propose the removal of *Napostygnus* from Metasarcinae based on male genitalia, which does not present the diagnostic character for the subfamily (Kury and Maury 1998; Kury and Pinto-da-Rocha 2007b), viz., a pair of spiny laterobasal sacs on the ventral plate. Another remarkable difference from Metasarcinae is the unarmed ventral pedipalpus femur. The combination of characteristics of penis and body morphology does not allow placing *Napostygnus bispinosus* in any other family of Laniatores which bear tarsal process on legs III–IV. The slightly swollen male basitarsus I is not equal to those of Manaosbiidae, and furthermore the genitalia is distinct, since it possesses a dorsal process. The ocularium resembles those of Gonyleptidae, Bourguyiinae, but the penis is not similar, because there is only a dorsal process and no ventral process. The aspect of the penis resembles those of Cranaidae (presence of dorsal process, ventral plate and setae shape) and therefore, we propose its transfer to this family. It is noteworthy to mention that the morphology of the body of *Napostygnus bispinosus* is somewhat distinct from the typical Cranaidae. We will not assign it to any subfamily, following the opinion of Orrico and Kury (2009) on the meaninglessness of current subfamily classifications in Cranaidae.

### Gonyleptidae Sundevall, 1833

Ampycinae Kury, 2003

#### Neopachyloides peruvianus

(Roewer, 1956) new comb., new subfamily assignment

http://species-id.net/wiki/Neopachyloides_peruvianus

Fig. 4

Ceropachylinus peruvianus Roewer, 1956: 440, fig. 19 (♂); Kury 2003: 158; (male holotype, Peru, near San Luis de Shuaro, 700–750 m, Koepcke leg, 17.III.1955, SMF 9791/1, examined).

##### Diagnosis.

**Figure 4.**
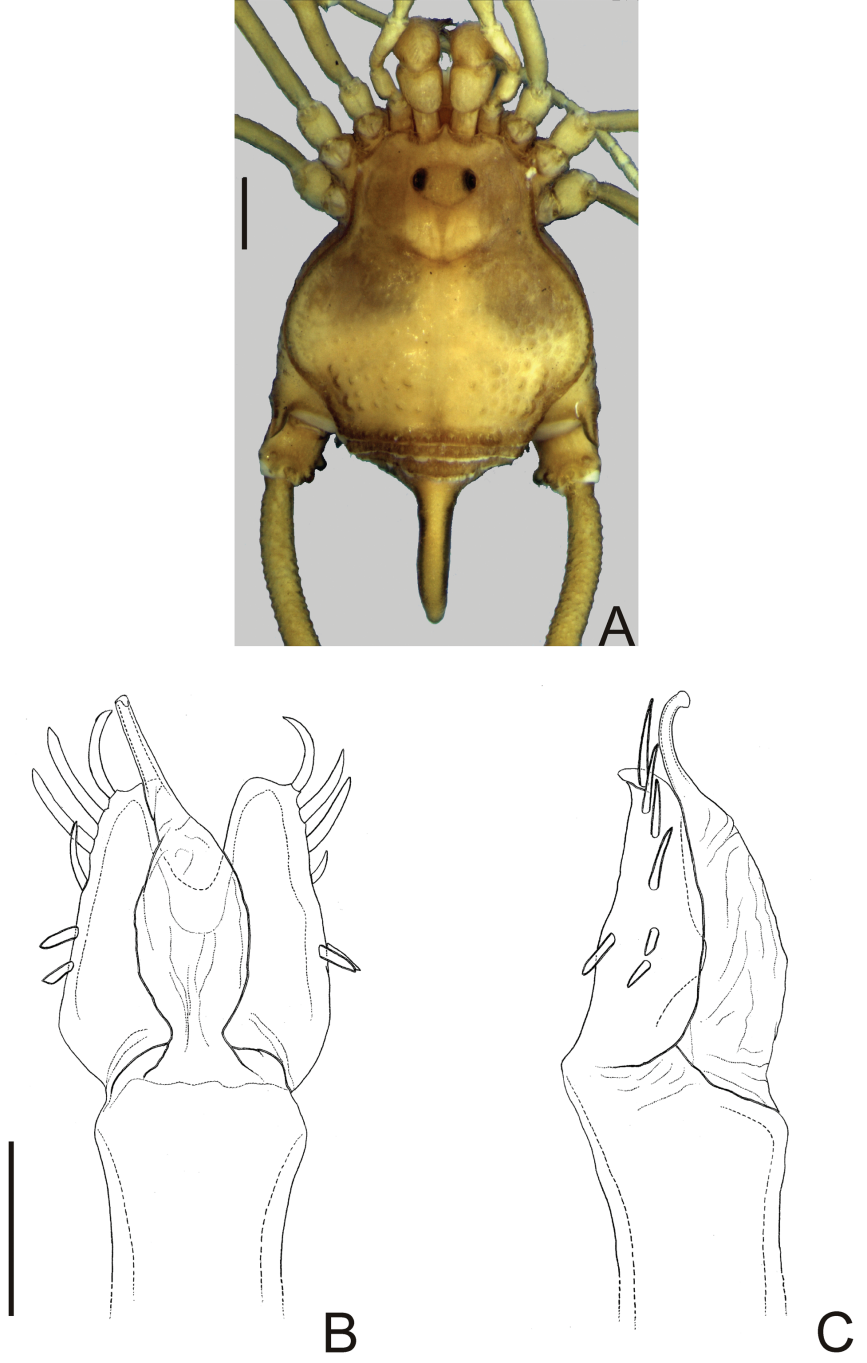
*Neopachyloides peruvianus* (Roewer) **comb. n.** Male (holotype): **A** habitus, dorsal view. Distal part of penis (holotype) **B** in dorsal view **C** ditto, left lateral view. **B, C** at same scale. Scale bars: **A** = 1 mm; **B** = 0.1 mm.

*Neopachyloides* was a hitherto monotypic genus that resembles Ampycinae genera with a paired armature on ocularium and free tergite III with a median, long spiniform apophysis asin *Ampycella* Roewer, 1929, *Ampycus* Simon, 1879, *Hutamaia* Soares & Soares, 1977 and *Sibollus* Roewer, 1929. *Neopachyloides* can be distinguished from these genera by the following combination of characteristics: scutal area III with a paramedian pair of enlarged, pointed tubercles, scutal area IV undivided and free tergite II unarmed. *Neopachyloides peruvianus* can be distinguished from *Neopachyloides spinipes* Roewer, 1913 by the dorsal scutum covered by granules and scutal areas I–II unarmed (Fig. 4A).

##### Description.

Penis (Fig. 4B–C; holotype): ventral plate with sub hexagonal shape, deep cleft on distal margin, 4 pairs of distal setae (distalmost curved and basalmost small), 3 pairs of basal setae straight. Glans very long (⅔ of ventral plate length), stylus smooth and curved dorsally, without dorsal and ventral processes.

##### Taxonomical note.

The assignment of *Neopachyloides peruvianus* to *Neopachyloides* is based on overall similarity and should be considered tentative. *Neopachyloides peruvianus* is the only Ampycinae which presents just one scutal area armed with a paramedian enlarged pair of tubercles (most genera present 3 scutal areas armed with a paramedian pair of enlarged tubercles, as *Ampycus*, *Hexabunus* and *Pirunipygus* or all of them unarmed). We preferred to place *Neopachyloides peruvianus* under *Neopachyloides* instead of proposing a new genus because monophyly of Ampycinae genera (most of them monotypic) are doubtful. See taxonomical note of *Pirunipygus paradoxus* for the reasons of the new placement of *Neopachyloides peruvianus*.

#### Pirunipygus

Roewer, 1936 new subfamily assignment

http://species-id.net/wiki/Pirunipygus

Pirunipygus Roewer, 1936: 341; Kury 2003: 187; (type species: *Pirunipygus paradoxus* Roewer, 1936, by monotypy).

##### Diagnosis.

**Figure 5.**
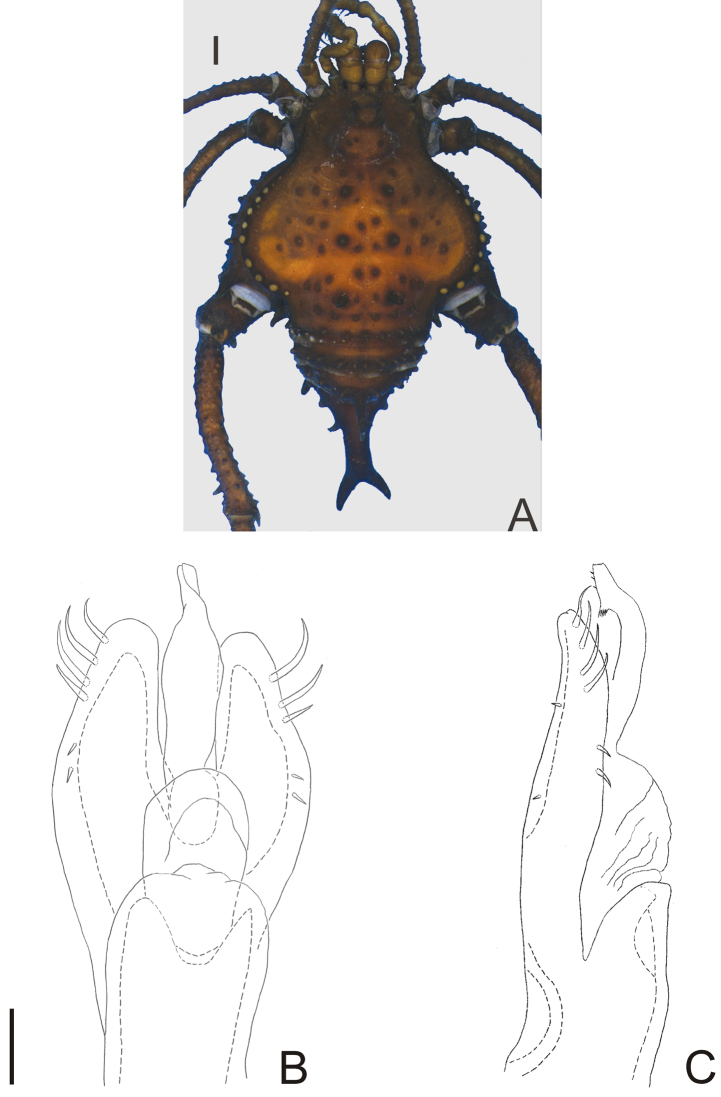
*Pirunipygus paradoxus* Roewer. Male (holotype): **A** habitus, dorsal view. Distal part of penis (holotype) **B** in dorsal view; **C** ditto, left lateral view. **B, C** at same scale. Scale bar: **A** = 1 mm. Scale bar: **B** = 0.1 mm.

*Pirunipygus* is a monotypic genus and resembles Ampycinae genera with a paramedian pair of enlarged tubercles on scutal areas I–II as *Ampycus* Simon, 1879, *Hexabunus* Roewer, 1913, *Neopachyloides* Roewer, 1913 and *Parahernandria* Goodnight & Goodnight, 1947. *Pirunipygus paradoxus* can be distinguished from these genera by the following combination of characters: ocularium with a median spiniform apophysis (Fig. 5A); dorsal scutum with secondary tubercles (sensu Maury 1992); scutal area III with a paramedian pair of spiniform apophyses; free tergite II with a median spiniform apophysis; free tergite III with three spiniform apophyses, the middle one largest and bifid.

##### Composition.

Monotypic.

#### Pirunipygus paradoxus

Roewer, 1936

http://species-id.net/wiki/Pirunipygus_paradoxus

Fig. 5

Pirunipygus paradoxus Roewer, 1936: 341, fig. 4 (♂); Kury 2003: 106; (male holotype, Peru, Junin, Tarma, SMF 6180/85, examined).

##### Description.

Penis (Fig. 5B–C; holotype): ventral plate sub hexagonal, deep U-cleft on distal margin, 3–4 distal pairs of setae (2–3 distalmost curved), 2 pairs of dorsal median setae of moderate size, 2 pairs of ventral median small setae. Stylus wide on basal ¾, apex twisted, with a patch of ventral subapical trichomes and some scattered apically. Glans without dorsal and ventral processes.

##### Taxonomical note.

Both *Neopachyloides peruvianus* and *Pirunipygus paradoxus* were formerly placed in Gonyleptidae, Pachylinae. These two species show the following diagnostic characteristics of Ampycinae (Kury 2003), viz., body coloration black, integument with huge rounded tubercles, stout apophysis on free tergite III, ventral plate of penis with deep U-shape cleft on distal margin, absence of dorsal and ventral processes of glans.

### Gonyleptinae Sundevall, 1833

#### Acanthogonyleptes

Mello-Leitão, 1922

http://species-id.net/wiki/Acanthogonyleptes

Acanthogonyleptes Mello-Leitão, 1922: 336; Kury 2003:137; DaSilva and Pinto-da-Rocha 2010: 626; (type species: *Acanthogonyleptes pulcher* Mello-Leitão, 1922, by original designation).
Centroleptes Roewer, 1943: 45; Kury 2003: 122; (type species: *Centroleptes flavus* Roewer, 1943, by monotypy). **Syn. n.**

##### Diagnosis.

As in Kury (2003: 137).

##### Taxonomical note.

The monotypic genus *Centroleptes* is a subjective synonym of *Acanthogonyleptes*, since the type examined material of *Centroleptes flavus* and *Acanthogonyleptes singularis* are identical and synonymized here.

##### Composition.

*Acanthogonyleptes alticola* (Mello-Leitão, 1922); *Acanthogonyleptes editus* (Roewer, 1943); *Acanthogonyleptes fallax* (Mello-Leitão, 1932); *Acanthogonyleptes fulvigranulatus* (Mello-Leitão, 1922); *Acanthogonyleptes marmoratus* (Mello-Leitão, 1940); *Acanthogonyleptes pictus* (Piza, 1942); *Acanthogonyleptes singularis* (Mello-Leitão, 1935); *Acanthogonyleptes soaresi* (Mello-Leitão, 1944); *Acanthogonyleptes variolosus* (Mello-Leitão, 1940).

#### Acanthogonyleptes singularis

(Mello-Leitão, 1935)

http://species-id.net/wiki/Acanthogonyleptes_singularis

Adelphobunus singularis Mello-Leitão, 1935: 392, fig. 19 (♂); Kury 2003: 137; (male holotype; Brazil, São Paulo, Ribeirão Pires, IBSP 17, examined).
Sphaerobunus singularis : Kury 2003: 139.
Acanthogonyleptes singularis : DaSilva and Pinto-da-Rocha 2010: 626.
Bocaina marmorata Piza, 1943: 46, fig. 5 (♂); Kury 2003: 139; (male holotype, Brazil, São Paulo, Serra da Bocaina, Fazenda Águas de Santa Rosa, MZSP 810, examined). Synonymy established by Soares and Soares (1987).
Centroleptes flavus Roewer, 1943: 45, fig. 50 (♀); Kury 2003: 122; (female holotype, Brazil, Santa Catarina, Seara, Nova Teutonia, SMF 6430/63, examined). **Syn. n.**

##### Material examined.

BRAZIL. São Paulo: São José do Barreiro (Serra da Bocaina, Fazendas Águas de Santa Rosa), female holotype of *Bocaina marmorata* Piza (MZSP 810); Ribeirão Pires, male holotype of *Adelphobunus singularis* Mello-Leitão (IBSP 17); São José do Barreiro, 1 ♂ (HEMS 761); Santo André, 1 ♂ & 1 ♀ (MZSP 21257). Santa Catarina: Seara (Nova Teutônia), ♀ holotype of *Centroleptes flavus* Roewer (SMF 6430/63).

##### Diagnosis.

*Acanthogonyleptes singularis* and *Acanthogonyleptes variolosus* can be distinguished from the remaining species of the genus by the presence of a median single elevation on scutal area III. Males of *Acanthogonyleptes singularis* can be distinguished from those of *Acanthogonyleptes variolosus* by the presence of a robust curved retrolateral apophysis on trochanter IV.

##### Taxonomical note.

The genus *Acanthogonyleptes* is a morphologically homogeneous group, probably representing a monophyletic group (see *Sphaerobunus* in Kury 2003). *Acanthogonyleptes singularis* and *Centroleptes flavus* bear a median single elevation on scutal area III. In addition, the morphology of female leg IV of both *Acanthogonyleptes singularis* and *Centroleptes flavus* are identical. The geographical records of *Acanthogonyleptes singularis* include localities in the Brazilian states of São Paulo and Rio de Janeiro. The only known specimen identified as *Centroleptes flavus* is the holotype, supposedly collected in Seara (Nova Teutônia), and therefore the locality cited by Roewer (1943) is considered wrong.

#### Geraeocormobius sylvarum

Holmberg, 1887

http://species-id.net/wiki/Geraeocormobius_sylvarum

Geraecormobius sylvarum Holmberg, 1887: 211, fig. unnumbered (♂); Kury 2003: 125; (1 male & 1 female syntypes, Argentina, Misiones, Santa Ana, near river Piraí Mirín, type depository unknown).
Discocyrtus serrifemur Roewer, 1943: 21, fig. 10 (♂); (1 male & 8 female syntypes, Brazil, Santa Catarina, Nova Teutônia, SMF 6193/104, examined). **Syn. n.**
Geraeocormobius sylvarum Acosta et al. 2007a: 303, figs 1–3 (map, ♂♀).

##### Other material examined.

Brazil. Paraná: Cachoeirinha, 2 ♂ & 5 ♀ (MNRJ 26912); Paranavaí, 6 ♂ & 7 ♀ (MNRJ 4732).

##### Taxonomical note.

Roewer (1943) placed *Discocyrtus serrifemur* in the Pachylinae based on the observation of pigmentation on posterior part of dorsal scutum, which he assumed erroneously to be the scutal area IV. Both sexes of type series of *Discocyrtus serrifemur* are identical to *Geraeocormobius sylvarum*. In general, females of Gonyleptinae do not show conspicuous diagnostic characters, which are mainly seen in male tegumentary ornamentation. Both male and female specimens of *Geraeocormobius sylvarum* preserved in 70% alcohol present a general black coloration with olive or yellowish lateral marks on the dorsal scutum, with white pedipalps. *Geraeocormobius sylvarum* could be confused with *Geraeocormobius rohri* (Mello-Leitão) and *Geraeocormobius salebrosus* (Roewer) because of its relatively larger body size, presence of large rounded tubercles on the scutal area III, general black coloration and geographical occurrence. However, *Geraeocormobius sylvarum* can be distinguished from them by the white pedipalps.

#### Gonyleptellus bimaculatus

(Sørensen, 1884) comb. n.

http://species-id.net/wiki/Gonyleptellus_bimaculatus

Gonyleptes bimaculatus Sørensen, 1884: 605; (female holotype, Brazil, ZMUC, examined from digital photos).
Paragonyleptes bimaculatus : Roewer 1913: 243.
Gonyleptes cancellatus Roewer, 1917: 127, fig. 26 (♂); (male holotype, Brazil, São Paulo, Santos, SMF 1320, examined). **Syn. n.**
Gonyleptellus cancellatus : Kury 2003: 126.
"Paragonyleptes" bimaculatus : Kury 2003: 121.

##### Material examined.

BRAZIL. Locality not specified further, female holotype of *Gonyleptes bimaculatus*, examined by digital photos (ZMUC). São Paulo: Santos, ♂ holotype of *Gonyleptes cancellatus* (SMF 1320); Bananal (Estação Ecológica do Bananal), A. Monteiro leg., IV.2004, 10 ♂ & 10 ♀ (MZSP 27804). Rio de Janeiro: Itatiaia (Parque Nacional do Itatiaia), Equipe Biota leg., 8–15.VI.2001, 3 ♂ & 1 ♀ (MZSP 21746); Terezópolis (Parque Nacional da Serra dos Órgãos, sede), R. Pinto-da-Rocha, S. Outeda-Jorge, C. Mattoni leg., 10.II.2007, 3 ♂ & 5 ♀ (MZSP 28223); Nova Friburgo (Mury, Debossan, 950 m), R.S. Bérnils & P. Labiak leg., 29.VII.1996, 6 ♂ & 3 ♀ (MZSP 15120).

##### Taxonomical note.

Sørensen (1884) described *Gonyleptes bimaculatus* from Brazil without any illustration. Roewer (1913) transferred it to *Paragonyleptes* Roewer, 1913. Kury (2003) synonymized the type species (*Gonyleptes bicuspidatus* Koch, 1839) of *Paragonyleptes* with *Collonychium bicuspidatum* Bertkau, 1880 and considered some species allocated in *Paragonyleptes* as Gonyleptinae *incertae sedis*, such as *Gonyleptellus bimaculatus*. The examination of *Gonyleptellus bimaculatus* from detailed photos of the holotype, which is reasonably well preserved, allowed us to recognize it as *Gonyleptellus cancellatus*, a well-known species from the Brazilian states of Rio de Janeiro and São Paulo (Serra da Bocaina mountain range). Therefore, *Gonyleptellus cancellatus* is a junior synonym of *Gonyleptellus bimaculatus*. The other species of *Gonyleptellus* is *Gonyleptellus bufoninus* (Mello-Leitão, 1949), known only by its original description, which lacks illustration and its type depository is unknown (Kury 2003).

#### Gonyleptes

Kirby, 1819

http://species-id.net/wiki/Gonyleptes

Gonyleptes Kirby, 1819: 450; Kury 2003: 126; (type species: *Gonyleptes horridus* Kirby, 1819, by subsequent designation, Roewer 1913).
Collonychium Bertkau, 1880: 108; Kury 2003: 122; (type species: *Collonychium bicuspidatum* Bertkau, 1880, by monotypy). **Syn. n.**

##### Diagnosis.

*Gonyleptes* is a genus composed of generally large species (dorsal scutum length longer than 8 mm), which males present femur IV with conspicuous armature and scutal areas I–III convex in lateral view. The penis presents ventral plate very convex and apex of ventral process serrate and semicircular. See taxonomical note below.

##### Taxonomical note.

*Gonyleptes* possesses 23 species and its habitus resembles other genera placed in Gonyleptinae. As Kury (2003) already suggested, the generic boundaries are not so clear cut. *Collonychium* is an objective synonym of *Gonyleptes* since both type species are synonymous. It can be only distinguished from related taxa, such as *Geraeocormobius* Holmberg, 1887, by a combination of unsatisfactory Roewerian characteristics. The composition of the genus below includes the changes proposed in this article.

**Composition.** *Gonyleptes acanthopus* (Quoy & Gaimard, 1824); *Gonyleptes armatus* Perty, 1833; *Gonyleptes atrus* Mello-Leitão, 1923, **revalidated**; *Gonyleptes barbiellinii* Mello-Leitão, 1932; *Gonyleptes calcaripes* (Roewer, 1917); *Gonyleptes curticornis* (Mello-Leitão, 1940); *Gonyleptes curvicornis* (Roewer, 1932), **revalidated**, **comb**. **n**.; *Gonyleptes fragilis* Mello-Leitão, 1923; *Gonyleptes gertschi* Soares & Soares, 1948; *Gonyleptes gonyleptoides* (Soares & Soares, 1945); *Gonyleptes granulatus* (Piza, 1940); *Gonyleptes horridus* Kirby, 1819; *Gonyleptes parcigranulatus* Soares & Soares, 1949; *Gonyleptes pectinatus* Koch, 1845; *Gonyleptes pectinipes* Roewer, 1917; *Gonyleptes perlatus* (Mello-Leitão, 1935), **comb**. **n**.; *Gonyleptes pseudogranulatus* Soares & Soares, 1946; *Gonyleptes pseudoguttatus* Giltay, 1928; *Gonyleptes pustulatus* Sørensen, 1884; *Gonyleptes recentissimus* Mello-Leitão, 1932; *Gonyleptes saprophilus* Mello-Leitão, 1922; *Gonyleptes vatius* Bertkau, 1880; *Gonyleptes viridisagittatus* Soares & Soares, 1945.

#### Gonyleptes horridus

Kirby, 1819

http://species-id.net/wiki/Gonyleptes_horridus

Figs 6A–D 7

Gonyleptes horridus Kirby, 1819: 452, pl. 22, fig. 16 (♂); Kury 2003: 128; (male holotype, Brazil, NHM 1863.41, examined from detailed photos).
Gonyleptes bicuspidatus Koch, 1839: 13; (2 females syntypes, Brazil, NHMW, not examined; 2 males & 2 females syntypes, Brazil, ZMB 905, not examined). **Syn. n.**
Collonychium bicuspidatum Bertkau, 1880: 108, pl. 2, fig. 39 (♀); (female nymph holotype, Brazil, Rio de Janeiro, Rio de Janeiro [Copacabana], ISNB, not examined). **Syn. n.**
Metagonyleptes hamatus Roewer, 1913: 213, fig. 89 (♂); (male holotype, Brazil, São Paulo, SMF 891, examined). **Syn. n.**
Paragonyleptes simoni Roewer, 1930: 379, fig. 12 (♂); (male holotype, Brazil, Santa Catarina, Serra Azul, SMF RII 1333, examined). **Syn. n.**
Gonyleptes borgmeyeri Mello-Leitão, 1932: 305, fig. 167 (♂); (male holotype, MNRJ, lost, Brazil, Rio de Janeiro, Petrópolis). **Syn. n.**
Gonyleptes curvicornis Mello-Leitão, 1932: 305, fig. 166 (♂); (male holotype, MNRJ, lost, Brazil, Rio de Janeiro, Itatiaia). **Syn. n.**
Gonyleptes melloleitaoi Kury & Alonso-Zarazaga, 2011: 54 (replacement name for *Gonyleptes curvicornis* Mello-Leitão, secondary homonym of *Weyhia curvicornis* Roewer).

##### Material examined.

BRAZIL. Locality not specified further, male holotype of *Gonyleptes horridus*, examined by digital photos (NHM 1863.41). São Paulo: São Paulo, male holotype of *Metagonyleptes hamatus* (SMF 891). Rio de Janeiro: Rio de Janeiro (Gávea), 1 male (MZSP 1370). Santa Catarina: (Serra Azul), male holotype of *Paragonyleptes simoni* (SMF RII 1333).

##### Diagnosis.

*Gonyleptes horridus* can be recognized by: the relatively larger size; anterior border of carapace smooth (without tubercles or spines in the corners); frontal hump on anterior border of carapace and ocularium with white small tubercles; prolateral apical apophysis of coxa IV long, with abrupt curvature backwards. *Gonyleptes horridus*, *Gonyleptes perlatus* and *Gonyleptes pustulatus* can be distinguished from all other species of *Gonyleptes* by the robust and bifid (two distal tips) retrolateral apical apophysis of coxa IV. Females of *Gonyleptes horridus* and *Gonyleptes perlatus* present dimorphic sulfur yellow spines on the free tergites, not present in females of *Gonyleptes pustulatus*. *Gonyleptes horridus* can be distinguished from *Gonyleptes perlatus* by the armature pattern of femur IV of the males (see note on *Gonyleptes perlatus* below). *Gonyleptes horridus* has relatively smaller tubercles on posterior part of the lateral margins of abdominal scutum than in *Gonyleptes perlatus*.

##### Description.

**Figure 6.**
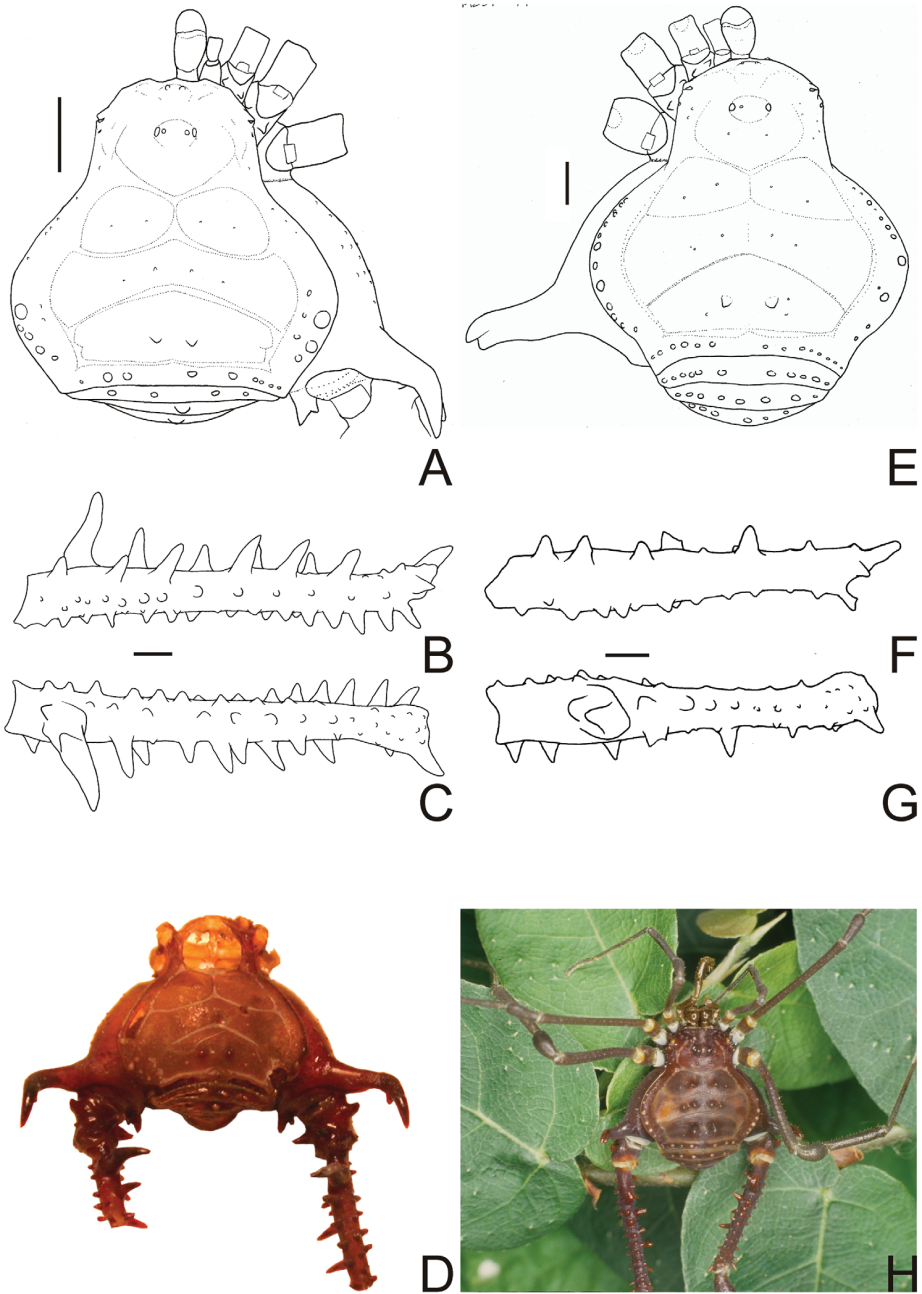
*Gonyleptes* spp. male dorsal habitus and femora IV: **A–D** *Gonyleptes horridus* Kirby (HEMS 103): **A** habitus, dorsal view **B** right femur IV ventral view **C** ditto, dorsal view **D** picture of the holotype (NHM 1863.41). **E–H** *Gonyleptes curvicornis* (Roewer) (MZSP 0871): **E** habitus, dorsal view **F** right femur IV ventral view **G** ditto, dorsal view **H** picture of a live specimen. **B, C** at same scale. **F, G** at same scale. Scale bars: 1 mm.

**Figure 7.**
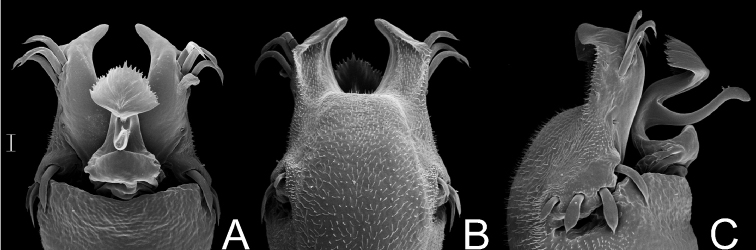
*Gonyleptes horridus* Kirby. Distal part of penis (MZSP 15496) **A** dorsal view **B** ditto, ventral view **C** ditto, left lateral view. Scale bar: 0.02 mm.

Dorsal scutum length 7–9 mm. Anterior margin of carapace without tubercles or spines. Median frontal hump on anterior margin of carapace and ocularium with a pair of tubercles on each one (Fig. 6A). Four scutal grooves delimiting three scutal areas. Scutal areas I, II with low white tubercles in the median region; III with high paramedian tubercles. Lateral margin of dorsal scutum with flattened white tubercles. Pedipalps approximately same length as dorsal scutum. Prolateral apophyses of coxa IV elongated, strongly curved backwards, dorsal branch longest. Retrolateral apophysis of coxa IV bifid. Femur IV straight, with dorsobasal apophyses and longitudinal rows of spines (Fig. 6B–C). Penis with typical Gonyleptinae-like pattern (Kury 1992b). Tarsal segmentation: 6(3); 10–12(3); 7–8; 8–9. Females with dimorphic cones (one yellowish huge median spine) on free tergites II and III. General coloration brown with white tubercles (Fig. 6D). Penis (Fig. 7A–C; MZSP 15496): ventral plate with a deep U-cleft on anterior margin (its distal tips convergent), 3 distal, 1 median and 4 basal pairs of setae, ventrally with a subdistal and a median pair of setae. Stylus sigmoid, with ventral subapical trichomes. Ventral process robust, apex as a flabellum with several small projections on distal margin.

##### Taxonomical note.

All synonyms given in Kury (2003) for *Gonyleptes horridus* refer, in fact, to *Gonyleptes curvicornis* (Roewer, 1913). The type material of *Gonyleptes curvicornis* Mello-Leitão, 1932 was lost, but the original descriptions and illustrations are sufficient to propose the present synonymy. The types of *Metagonyleptes hamatus* Roewer and *Paragonyleptes simoni* Roewer are identical to that of *Gonyleptes horridus* Kirby. The record of *Gonyleptes horridus* in Santa Catarina (type locality of *Paragonyleptes simoni)* is dubious. The synonymy of *Gonyleptes bicuspidatus* with *Gonyleptes horridus* is here proposed based on the reasoning of Kury (2003), who considered this nominal species as synonym of *Collonychium bicuspidatum*. In turn, *Collonychium bicuspidatum* is clearly a synonym of *Gonyleptes horridus*.

#### Gonyleptes curvicornis

(Roewer, 1913) revalidated, comb. n.

http://species-id.net/wiki/Gonyleptes_curvicornis

Figs 6E–H 8

Weyhia curvicornis Roewer, 1913: 193, fig. 80 (♂); Kury 2003: 128; (holotype male, Brazil, São Paulo, SMF 979, examined).
Gonyleptes lacrimosus Mello-Leitão, 1932: 294, fig. 148 (♂); (male holotype, Brazil, Rio de Janeiro (Floresta da Tijuca), MNRJ 11789, not examined).
Mendesius albipunctatus Roewer, 1943: 41, pl. 5, fig. 45 (♀); (female holotype, Brazil, Mendes, SMF 5389/47).

##### Material examined.

BRAZIL. São Paulo: Locality not specified further, male holotype (SMF 979); São Paulo (Chácara Dr. J. L. Lane), male (MZSP 0871). Rio de Janeiro: Rio de Janeiro (Grajaú), 1 male (MZSP 1117).

##### Diagnosis.

*Gonyleptes curvicornis* resembles *Gonyleptes horridus* (see diagnosis above) but can be distinguished by the relatively shorter prolateral apical apophysis of coxa IV, lacking the abrupt backwards curvature (Fig. 6E). The retrolateral apical apophysis of coxa IV is reduced and not bifid as in *Gonyleptes horridus*. The male femur IV has a different pattern of armature (Fig. 6F–G) compared to *Gonyleptes horridus*. Carapace with a pair of tubercles on the posterior area (posterior to ocularium). Females of *Gonyleptes curvicornis* do not present dimorphic sulfur yellow spines on the free tergites.

##### Description.

**Figure 8.**
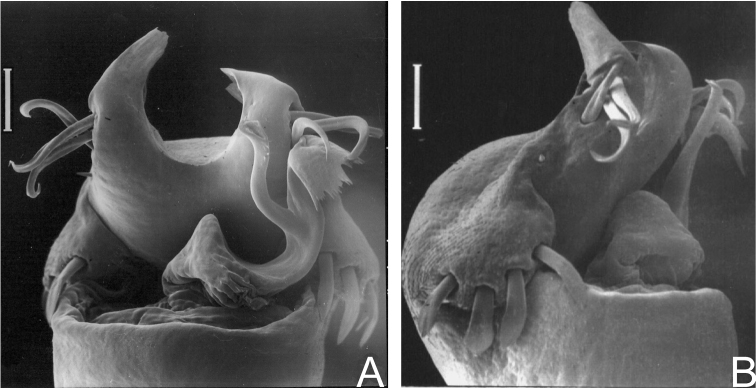
*Gonyleptes curvicornis* (Roewer). Distal part of penis (MZSP 1117) **A** dorsal view **B** ditto, left lateral view. Scale bars: 0.05 mm.

Penis (Fig. 8A–B; MZSP 1117): ventral plate sub hexagonal, with a deep U-cleft on distal margin (its distal tips convergent), 3 distal, 1 median, 4 basal pairs of setae (distal ones curved, median ones shortest). Stylus sigmoid, with ventral subapical trichomes. Ventral process robust, apex flabelliform with several projections of varying sizes on distal margin.

##### Taxonomical note.

Roewer’s (1913; 1923) redescriptions of *Gonyleptes horridus* and *Weyhia curvicornis* are the same. Following *Gonyleptes horridus sensu* Roewer, Soares and Soares (1988) considered *Weyhia curvicornis* as its junior synonym. However, as seen above, such redescription of *Gonyleptes horridus* was mistaken and, therefore, *Weyhia curvicornis* can no longer be considered as a junior synonym. *Weyhia* was synonymized with *Geraeocormobius* by Soares and Soares (1949). The Gonyleptinae genera definition is still unsatisfactory, but the placement of *Weyhia curvicornis* in *Gonyleptes* is more reasonable than its placement in *Geraeocormobius*, due to morphological similarities shared with *Gonyleptes horridus*, such as white tubercles on frontal hump on anterior margin of dorsal scutum, ocularium (Fig. 6H) and lateral borders of abdominal scutum convex and abdominal scutum itself convex.

#### Gonyleptes atrus

Mello-Leitão, 1923 revalidated

http://species-id.net/wiki/Gonyleptes_atrus

Figs 9A–B 10

Gonyleptes atrus Mello-Leitão, 1923: 140; Kury 2003: 129; (male syntype, Brazil, Itatiaia, state of Rio de Janeiro or Minas Gerais, MNRJ 1462, examined; 3 males & 3 females syntypes, MZSP 42).
Weyhia brieni Giltay, 1928: 83; (male holotype; Brazil, Itatiaia; ISNB; not examined). **Syn. n.**
Geraeocormobius jimi Soares & Soares, 1974: 601, figs 8–25 (♂♀); (male holotype and 1 female paratype; Brazil, Rio de Janeiro; Itatiaia; J. Jim et al. leg, 28.XI.1977; HSPC 447; 2 males paratypes; same locality; A. Peracchi & E. Izecksohn leg, 9.IV.1966; HSPC 463; examined). **Syn. n.**

##### Material examined.

BRAZIL. Minas Gerais: Lambari (Parque Estadual Nova Baden), (MZSP 32513); Delfim Moreira (MZSP 29393); Itamonte (MZSP 21251); Poços de Caldas (MZSP 29366). Rio de Janeiro: Itatiaia, syntypes of *Weyhia bisignata* (MNRJ 27321). São Paulo: Campos do Jordão (MZSP 21251).

##### Diagnosis.

**Figure 9.**
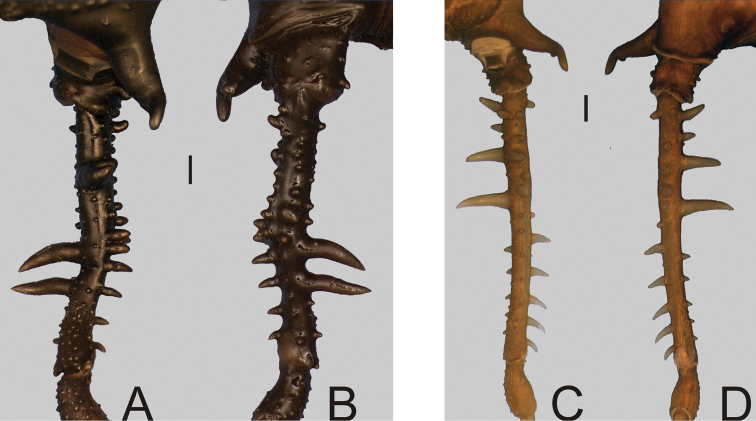
*Gonyleptes* spp. male femora IV: **A, B** *Gonyleptes atrus* Mello-Leitão (MZSP 32513): **A** right femur IV dorsal view **B** ditto, ventral view. **C, D** *Gonyleptes saprophilus* Mello-Leitão (MZSP 9996): **C** right femur IV dorsal view **D** ditto, ventral view. **A, B** at same scale. **C, D** at same scale. Scale bars: 1 mm.

Dorsal scutum length 8–10 mm. Corners of anterior margin of carapace smooth. Median frontal hump on anterior margin of carapace and ocularium with one pair of tubercles each one. Posterior region of carapace with 2 median tubercles. Mesotergum densely covered by tubercles. Scutal areas I–III each with a pair of median enlarged tubercles (III with largest and round ones). Prolateral apical apophysis of male coxa IV slightly directed backwards, retrolateral apophysis of male coxa IV absent or very reduced (Fig. 9A–B). Dorsobasal apophysis of male femur IV robust, curved and retrolaterally oriented. Ornamentation of male femur IV variable, normally with two prolateral spines.

##### Description.

**Figure 10.**
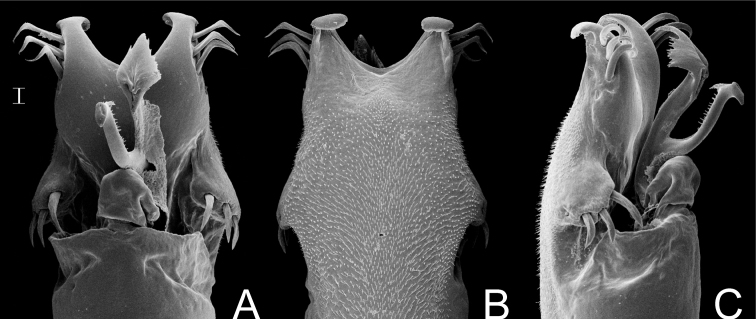
*Gonyleptes atrus* Mello-Leitão. Distal part of penis (MZSP 29393) **A** dorsal view **B** ditto, ventral view **C** ditto, left lateral view. Scale bar: 0.02 mm.

Penis (Fig. 10A–C, MZSP 29393): ventral plate with 3 distal and 3 basal pairs of large setae, 1 median pair of short setae. Stylus with ventral trichomes. Ventral process with a triangular flabellum, with digitiform ventral median projection.

##### Taxonomical note.

*Gonyleptes atrus* was synonymized, without further remarks, with *Gonyleptes saprophilus* by Kury (2003) in his catalogue of Laniatores of the New World. However, the study of type material of both species revealed no overlap of some diagnostic characteristics. *Gonyleptes atrus* is relatively larger than *Gonyleptes saprophilus*. Both species present different patterns of ornamentation on male femur IV. *Gonyleptes atrus* shows a sigmoid femur IV with robust dorsal subasal apophysis and two remarkable retrolateral apophyses (Fig. 9A–B), while *Gonyleptes saprophilus* shows a straight femur, reduced dorsal subasal apophysis, one remarkable retrolateral apophysis and three to five dorsal spines on anterior half of the femur IV (Fig. 9C–D). Specimens of *Geraeocormobius jimi* and *Gonyleptes atrus* are identical. The description of *Weyhia brieni* is sufficiently clear to consider this species synonymy of *Gonyleptes atrus*, even without the examination of the type material. Only *Gonyleptes itatiayae* Mello-Leitão, *Weyhia bisignata* Mello-Leitão and *Gyndesops pretiosus* Mello-Leitão, and all its combination, remain in the synonymic list of *Gonyleptes saprophilus*. *Gonyleptes atrus* occurs in sympatry with *Gonyleptes saprophilus* in almost all of its distribution range.

#### Gonyleptes fragilis

Mello-Leitão, 1923

http://species-id.net/wiki/Gonyleptes_fragilis

Fig. 11

Gonyleptes fragilis Mello-Leitão, 1923: 127; Kury 2003: 127; (female holotype, Brazil, São Paulo, Santo André, Alto da Serra, MZSP 56, examined).
Mendesius guttatus Roewer, 1952: 57; (female holotype, Brazil, SMF RII 10311/72, examined).
Gonyleptes banana Kury, 2003: 127 (replacement name for *Mendesius guttatus* Roewer, 1952, preoccupied by *Gonyleptes guttatus* Roewer, 1917). **Syn. n.**

##### Diagnosis.

*Gonyleptes fragilis* resembles *Gonyleptes horridus* and *Gonyleptes curvicornis* because of the overall similarities of the features of the dorsal scutum. *Gonyleptes fragilis* can be distinguished from the other two species by the ocularium with a pair of spines (tubercles in *Gonyleptes horridus* and *Gonyleptes curvicornis*) and the pale yellowish spots around the tubercles on abdominal scutum.

##### Description.

**Figure 11.**
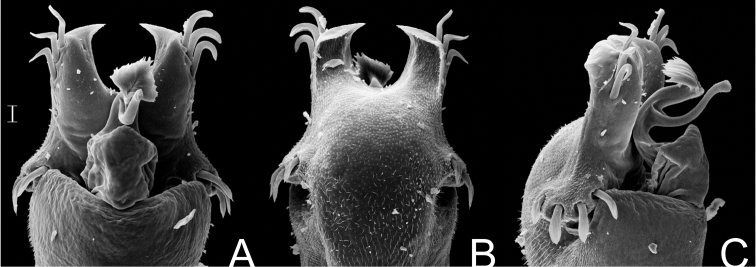
*Gonyleptes fragilis* Mello-Leitão. Distal part of penis (MZSP 30764) **A** dorsal view **B** ditto, ventral view **C** ditto, left lateral view. Scale bar: 0.02 mm.

Penis (Fig. 11A–C): ventral plate inflated in the middle region, with a deep U-cleft on anterior margin (its distal tips convergent), 3 distal pairs of long, curved setae, 1 median pair of short setae, 4 basal pairs of spatulate setae. Glans sac slightly projected distally, with similar projection to a dorsal process. Stylus sigmoid, smooth. Ventral process robust, apex flabelliform with several small projections on distal margin.

##### Taxonomical note.

Soares and Soares (1988) proposed the new combination *Gonyleptes guttatus* (Roewer, 1952) for *Melloleitaniella guttatus*, making it a homonym of *Gonyleptes guttatus* Roewer, 1917 [= *Melloleitaniella guttata* (Roewer, 1917)]. Kury (2003) renamed the species as *Gonyleptes banana*, based on the Roewer’s citation about the peculiar collection site of the type specimen, a banana shipment for export. The female holotype of *Mendesius guttatus* matches exactly the female holotype of *Gonyleptes fragilis*. Both males and females of *Gonyleptes fragilis* present a brown general coloration with some pale yellowish spots around the tubercles of the mesotergum, a diagnostic characteristic for this species. In addition, the occurrence area of *Gonyleptes fragilis* includes the Vale do Ribeira and southern coast of São Paulo (Kury 2003), an important area known for producing banana for exportation in the 1940’s and 1950’s.

#### Gonyleptes perlatus

(Mello-Leitão, 1935) comb. n.

http://species-id.net/wiki/Gonyleptes_perlatus

Moojenia perlata Mello-Leitão, 1935: 384, fig. 13 (♂); (2 males syntypes, Brazil, Minas Gerais, Viçosa, MNRJ 1577, examined)
Collonychium perlatum : Kury 2003: 123.

##### Diagnosis.

*Gonyleptes perlatus* is very similar to *Gonyleptes horridus* (according to Kury 2003) but can be distinguished by the relatively larger tubercles on lateral margin of the dorsal scutum and the absence of a robust prolateral row of apophysis on male femur IV (present in *Gonyleptes horridus*), although this article might present variable armature.

##### Taxonomical note.

We propose the allocation of this species in *Gonyleptes* because it is very similar to *Gonyleptes horridus* (see comments in Kury 2003), including the typical sexual dimorphism. According Kury (2003), both species are allopatric.

#### Gonyleptes pustulatus

Sørensen, 1884

http://species-id.net/wiki/Gonyleptes_pustulatus

Fig. 12

Gonyleptes pustulatus Sørensen, 1884: 603, Kury 2003: 128; (male holotype, Brazil, ZMUC, examined from detailed photo).
Gonyleptes guttatus Roewer, 1917: 125, fig. 25 (♂); Kury 2003: 128; (male holotype, Brazil, São Paulo, Santos, SMF 1321, examined). **Syn. n.**

##### Material examined.

BRAZIL. Locality not specified further, male holotype of *Gonyleptes pustulatus*, examined from photo (ZMUC). São Paulo: Santos, male holotype of *Gonyleptes guttatus* (SMF 1321). Rio de Janeiro: Casimiro de Abreu, 5 ♂ & 6 ♀ (MNRJ 17456).

##### Diagnosis.

**Figure 12.**
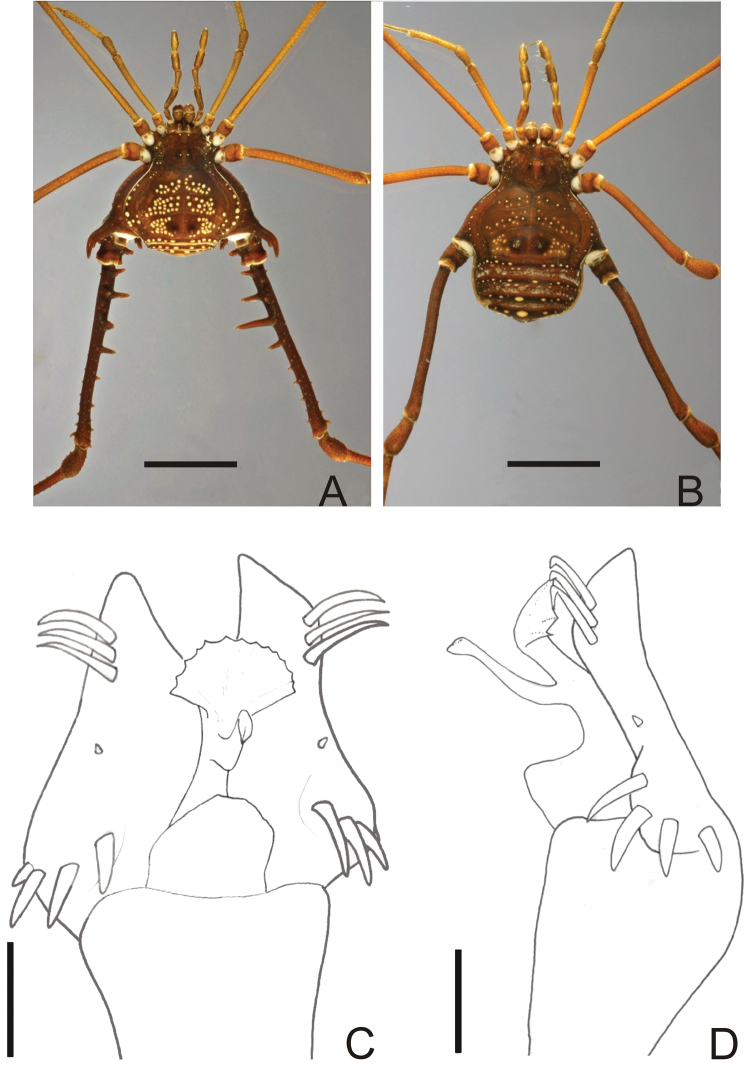
*Gonyleptes pustulatus*Sørensen. Male (MNRJ 17456): **A** habitus in dorsal view. Female (MNRJ 17456): **B** habitus in dorsal view. Distal part of penis (MNRJ 17456) **C** in dorsal view **D** ditto, right lateral view. Scale bars: **A–B** = 1.0 mm; **C–D** = 0.01 mm.

*Gonyleptes pustulatus* (Fig. 12A–B) superficially resembles *Gonyleptes bimaculatus* because of the conspicuous white patches on mesotergum. Males of *Gonyleptes pustulatus* can be distinguished from *Gonyleptes bimaculatus* by: femur IV armed (unarmed in *Gonyleptes bimaculatus*) and presence of a bifid retrolateral apical apophysis on coxa IV.

##### Description.

Penis (Fig. 12C–D; MNRJ 17456): ventral plate with slightly inflated middle region, a deep distal U-cleft on anterior margin (its distal tips are divergent), 3 distal pairs of long, curved setae, 1 median pair of short setae, 4 basal pairs of spatulate setae. Stylus slightly straight, with apex slightly swollen. Ventral process robust, apex flabelliform with serrated and rounded distal margin.

##### Taxonomical note.

*Gonyleptes pustulatus* is a remarkable species by body color pattern. It was described without any illustration. Probably this was the reason that kept this species virtually unknown and led Roewer to describe *Gonyleptes guttatus*.

#### Liogonyleptoides minensis

(Piza, 1946)

http://species-id.net/wiki/Liogonyleptoides_minensis

Chaquesia minensis Piza, 1946: 365, fig. 1 (♂); (2 males & 3 females syntypes; Brazil, Minas Gerais, Cachoeira do Pajeú; MZLQ A0053; examined).
Liogonyleptoides minensis : Kury 2003: 130.
Currala bahiensis Soares, 1972: 56, figs 1–4, 14–15 (♂♀); Kury 2003: 123; (male holotype and 1 female paratype; Brazil, Bahia, Maracás; MNRJ 5268; examined). **Syn. n.**

##### Taxonomical note.

Comparisons between the type material of *Currala bahiensis* and *Liogonyleptoides minensis* allowed us to conclude that they are synonymous. *Liogonyleptoides minensis* is a species that can be easily diagnosed among gonyleptines by the relatively larger size, body dorsally rounded, without tubercles on corners, presence of scutal area IV; femur III curved (instead of sigmoid); male femur IV with a long, robust dorsobasal apophysis and a prolateral one that is curved dorsally on distal third. The genus *Currala* is now composed only by its type species *Currala spinifrons* Roewer, 1927.

#### Mischonyx

Bertkau, 1880

http://species-id.net/wiki/Mischonyx

Mischonyx Bertkau, 1880: 106; Kury 2003: 132; (type species: *Mischonyx squalidus* Bertkau, 1880, by monotypy).
Eugonyleptes Roewer, 1913: 219; Kury 2003: 123; (type species: *Gonyleptes scaber* Kirby, 1819, by monotypy). **Syn. n.**
Gonazula Roewer, 1930: 417; Kury 2003: 126; (type species: *Gonazula gibbosa* Roewer, 1930, by monotypy). **Syn. n.**

##### Diagnosis.

*Mischonyx* resembles *Currala* Roewer, 1927 and *Schenkelibunus* Strand, 1932, which are gonyleptine of relatively smaller size (about 5 mm of dorsal scutum length). *Mischonyx* can be distinguished from those genera by the combination of the following characters: anterior border of the carapace with robust spines; frontal hump on anterior margin of carapace and ocularium with a pair of spines; scutal areas I–III with a paramedian pair of tubercles, those on I–II elliptical and widest pair on scutal area III; lateral margin of the dorsal scutum with large white tubercles (in specimens preserved in 70% ethanol).

##### Taxonomical note.

According DaSilva and Pinto-da-Rocha (2010), *Mischonyx* is phylogenetically close to Hernandariinae. It is probable that due to the Roewerian classification, some species which should be in *Mischonyx* is placed elsewhere in Gonyleptinae. Therefore, a comprehensive revision of the subfamily is needed.

##### Composition.

*Mischonyx anomalus* (Mello-Leitão, 1936); *Mischonyx antiquus* (Mello-Leitão, 1934); *Mischonyx cuspidatus* (Roewer, 1913); *Mischonyx fidelis* (Mello-Leitão, 1931); *Mischonyx insulanus* (Soares, 1972); *Mischonyx intermedius* (Mello-Leitão, 1935); *Mischonyx kaisara* Vasconcelos, 2004; *Mischonyx poeta* Vasconcelos, 2005; *Mischonyx processigerus* (Soares & Soares, 1970); *Mischonyx squalidus* Bertkau, 1880 and *Mischonyx sulinus* (Soares & Soares, 1947).

#### Mischonyx scaber

(Kirby, 1819) comb. n.

http://species-id.net/wiki/Mischonyx_scaber

Gonyleptes scaber Kirby, 1819: 453; (3 males & 1 female syntypes; Brazil; NHM 1863.41; examined from detailed photo).
Eugonyleptes scaber : Roewer, 1913: 219; Kury 2003: 123.
Xundarava holacantha Mello-Leitão, 1927: 20; (female holotype; Brazil, Rio de Janeiro, Niteroi; MNRJ 1469; examined).**Syn. n.**
Mischonyx holacanthus : Kury 2003: 133.

##### Material examined.

BRAZIL. Locality not specified further, 3 males & 1 female syntypes (NHM 1863.41). Rio de Janeiro: Niterói, female holotype of *Xundarava holacantha* (MNRJ 1469); ditto, male holotype of *Weyhia absconsa* (MNRJ 1483).

##### Diagnosis.

*Mischonyx scaber* resembles *Mischonyx cuspidatus* and *Mischonyx poeta* because of armature of the male femur IV: presence of a dorsobasal apophysis relatively short and curved; one retrolateral apophysis on distal third; and a row of high, acuminated tubercles increasing in size from the base to distal third. *Mischonyx scaber* can be distinguished from *Mischonyx cuspidatus* by the presence of a short gap between the retrolateral row and the retrolateral apophysis on femur IV (the retrolateral row is continuous to the retrolateral apophysis on femur IV in *Mischonyx cuspidatus*). *Mischonyx scaber* can be distinguished from *Mischonyx poeta* by the absence of enlarged tubercles (present in *Mischonyx poeta*) on the posterior part of the lateral margin of the dorsal scutum.

##### Taxonomical note.

*Mischonyx scaber* was described from Brazil, however, many authors, including Kury (2003), cited other countries as its type locality. It is clearly written “Brazil” on the label of the *Gonyleptes scaber* holotype. The only other known specimens of this species are the types of *Xundarava holacantha* and *Weyhia absconsa* (both in poor condition). The precise distribution records are Rio de Janeiro (Ilha do Governador) and Niterói.

#### Mischonyx cuspidatus

(Roewer, 1913)

http://species-id.net/wiki/Mischonyx_cuspidatus

Ilhaia cuspidata Roewer, 1913: 221; (male holotype; Brazil, Rio de Janeiro, Ilha Grande, SMF 900; examined).
Mischonyx cuspidatus : Kury 2003: 133.
Gonazula gibbosa Roewer, 1930: 418, fig. 32 (♂); Kury 2003: 126; (male holotype; Brazil, Santa Catarina, Serra Azul; SMF 1328; examined). **Syn. n.**

##### Material examined.

BRAZIL. Rio de Janeiro: Ilha Grande, male holotype (SMF 900); Macaé, 16 ♂ & 6 ♀ (MNRJ 4601). Santa Catarina: Serra Azul, male holotype (SMF 1328); Gaspar, 1 ♂ (MNRJ 11584).

##### Diagnosis.

*Mischonyx cuspidatus* resembles *Mischonyx poeta* and *Mischonyx scaber* (see diagnosis of *Mischonyx scaber*). *Mischonyx cuspidatus* can be distinguished from them by the median high, acuminated tubercle on the free tergites I–III.

##### Taxonomical note.

The type locality of *Gonazula gibbosa*, Serra Azul (in the state of Santa Catarina), is an unknown toponym from where seven species are recorded (see Kury 2003). As some of these species were rediscovered in Serra dos Órgãos (in the state of Rio de Janeiro), we believe that the opilionids from Serra Azul were mislabeled, although *Mischonyx cuspidatus* is a synanthropic gonyleptid (Mestre and Pinto-da-Rocha 2004) with one of the largest geographical distributions known among Neotropical harvestmen (see records in Kury 2003).

#### Megapachylus grandis

Roewer, 1913

http://species-id.net/wiki/Megapachylus_grandis

Fig. 13

Megapachylus grandis Roewer, 1913: 124, fig. 56 (♂); Kury 2003: 131.
Metapachyloides almeidai Soares & Soares, 1946: 317, fig. 2 (♂); Kury 2003: 176; (male holotype; Brazil, Batea, São Paulo, Lane & Soares leg., V.1943; MZSP 883; examined) **Syn. n.**

##### Description.

**Figure 13.**
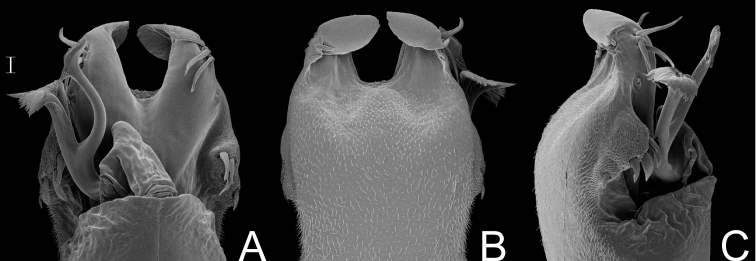
*Megapachylus grandis* Roewer. Distal part of penis (MZSP16837) **A** in dorsal view **B** ditto, ventral view **C** ditto, left lateral view. Scale bar: 0.02 mm.

Penis (Fig. 13A–C; MZSP16837): ventral plate with slightly convergent lateral sides deep U-cleft on distal margin, its apex folded ventrally, 3 distal pairs of large setae, slightly curved at apex, 1 median pair of short setae and, 4 basal pairs of spatulate setae, directed frontwards. Glans with dorsal process. Stylus thin, long, sigmoid, with subapical ventral trichomes. Ventral process shaft folded ventrally, apex flabelliform, with serrate distal margins and long distal tip.

##### Taxonomical note.

The male of the species described by Soares & Soares shows a moderate development of armature of leg IV but the number and arrangement of tubercles are perfectly coincident with those of *Megapachylus grandis*. It could be a beta male, although an extensive population study should be conducted to confirm this.

#### Parampheres

Roewer, 1913

http://species-id.net/wiki/Parampheres

Parampheres Roewer, 1913: 345; (type species: *Parampheres pectinatus* Roewer, 1913, by monotypy).
Metapachyloides Roewer, 1917: 120; (type species: *Metapachyloides rugosus* Roewer, 1917, by monotypy) **Syn. n.**

##### Diagnosis.

*Parampheres* resembles *Acanthogonyleptes*, *Adhynastes* Roewer, 1930 and *Deltaspidium* Roewer, 1927 by the presence of a more elongated prolateral apical apophysis on male coxa IV (greater length/basal diameter ratio of the apophysis) than that in *Gonyleptes* and related Gonyleptinae genera. *Parampheres* can be distinguished from *Adhynastes* and *Deltaspidium* by the scutal area III with tubercles (instead of spines). *Parampheres* can be distinghished from A*canthogonyleptes* by the absence of a clearly defined dorsobasal apophysis on the male femur IV. *Parampheres* species bear a row of apophyses on the dorsal basal area of the femur IV.

##### Taxonomical note.

*Metapachyloides* is a monotypic genus and its type species is junior synonym of *Parampheres tibialis* Roewer, 1917.

#### Parampheres tibialis

Roewer, 1917

http://species-id.net/wiki/Parampheres_tibialis

Parampheres tibialis Roewer, 1917: 144, fig. 37 (♂); (male holotype; Brazil, São Paulo, Santos; SMF 1330; examined).
Metapachyloides rugosus Roewer, 1917: 121, fig. 22 (♀); (female holotype; Brazil, São Paulo, Santos; SMF 1322; examined). **Syn. n.**

##### Taxonomical note.

The monotypic genus *Metapachyloides* was formerly placed in Gonyleptidae, Pachylinae. Instead of a male, as stated in the original description, the holotype of *Metapachyloides rugosus* is a female, and its general aspect is similar to that of *Parampheres* Roewer, 1913. Surprisingly, in the same year, Roewer described *Parampheres tibialis* in Gonyleptinae, based on a male collected in the same locality (Santos), the most important harbor in Brazil. Roewer described many harvestmen species indicating Santos as their type locality, but those were never collected again there or in the area of endemism to which Santos belongs (Serra do Mar of São Paulo, see Pinto-da-Rocha et al. 2005 for more details). We conclude that *Parampheres tibialis*, as well as many other species supposedly collected in Santos were probably mislabeled.

#### Parapachyloides uncinatus

(Sørensen, 1879)

http://species-id.net/wiki/Parapachyloides_uncinatus

Fig. 14

Gonyleptes uncinatus Sørensen, 1879: 214, figs 1–5, 9–10, 14, 18, 22–26, 28–29 (♂♀); (syntypes males & females, ZMUC, type locality not specified, see Kury 2003; examined from detailed photos).
Parapachyloides uncinatus : Kury 2003: 186.
Goyazella armata Mello-Leitão, 1931: 120, fig. 1 (♀); (female holotype; MNRJ 11373; Brazil; Goiás; Chapada dos Veadeiros; Blases leg., examined). syn. n.
Parapachyloides armatus : B. Soares 1944b: 164; Kury 2003: 186; Kury et al. 2010: 566.

##### Material examined.

BRAZIL. São Paulo: Porto Cabral, 4 ♂ (MZSP 787); Usina Hidrelétrica de Rosana, 2 males & 2 females (MZSP 14571). Mato Grosso do Sul: Taquaruçu, 2 ♂ & 1 ♀ (MZSP 19113); ditto, (Canal do Rio Baía), 1 ♂ (MZSP 22084).

##### Description.

**Figure 14.**
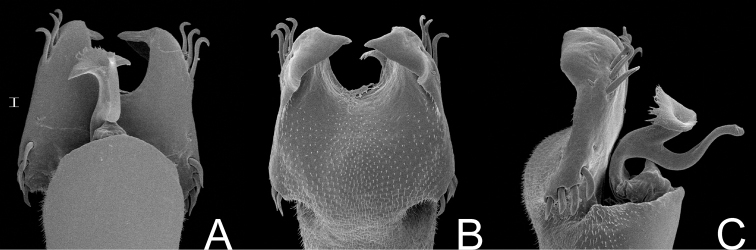
*Parapachyloides uncinatus* (Sørensen). Distal part of penis (MZSP 14571) **A** in dorsal view **B** ditto, ventral view **C** ditto, left lateral view. Scale bar: 0.01 mm.

Penis (Fig. 14A–C; MZSP 14571): ventral plate with the lateral sides slightly convergent, deep U-cleft on distal margin, its apex folded ventrally and convergent, 3 distal pairs of setae curved on apex, 1 pair of short median setae, 4 basal pairs of setae arranged in a row, 2 pairs of ventral distal short setae. Glans with small dorsal process. Stylus slender, sigmoid, smooth. Ventral process apex flabelliform with serrate distal margin.

##### Taxonomical note.

Males of this species show polymorphism in the posterior tubercle on the lateral margin of dorsal scutum (from low, small and blunt to high, enlarged, curved and pointed apex), posterior margin of dorsal scutum (from low tubercles to two high tubercles) and armature of free tergite I (with one large median tubercle without enlarged lateral tubercles, or with 2–4 high lateral tubercles); retrolateral basal tubercle on male femur IV of same width as femur to 1.5 times wider, short (1.3 times femur width) to long (3.2 ×).

#### Schubartesia singularis

B. Soares, 1944a

http://species-id.net/wiki/Schubartesia_singularis

Fig. 15

Schubartesia singularis B. Soares, 1944a: 34, fig. 1 (♂); Kury 2003: 192; Kury et al. 2010: 566; (male holotype, Brazil, Bahia, Os Gerais, Rio Branco Valley, MZSP, examined).

##### Material examined.

BRAZIL. Bahia: (Os Gerais, Rio Branco Valley), male holotype of *Schubartesia singularis* (MZSP). Minas Gerais: Januária (Parque Nacional Cavernas do Peruaçu), 1 ♂ & 1 ♀ (MZSP 29075); ditto, 2 ♂ (MZSP 29839).

##### Description.

**Figure 15.**
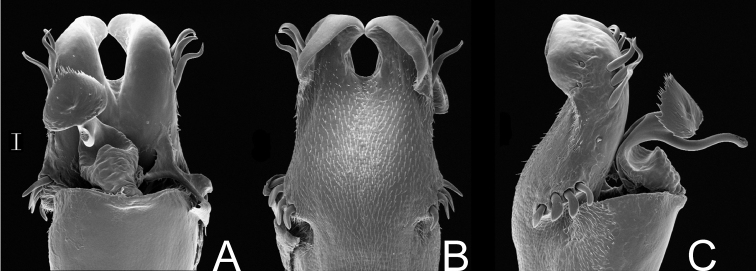
*Schubartesia singularis* B. Soares. Distal part of penis (MZSP 29075) **A** in dorsal view **B** ditto, ventral view **C** ditto, left lateral view. Scale bar: 0.02 mm.

Penis (Fig. 15A–C; MZSP 29075): ventral plate with the sides subparallel, with deep cleft on distal margin (U-cleft in ventral view and V-cleft in dorsal view; its apex convergent), 3 pairs of long helycoidal setae, 2 pairs of ventral distal short setae, 1 pair of median short setae, 4 pairs of spatulate, large setae arranged in an oblique row in lateral view, basal lobe short and projected dorsad. Stylus sigmoid, smooth. Ventral process shaft short and wide, apex flabelliform with slightly serrated distal margin.

##### Taxonomical note. 

*Schubartesia singularis* and *Parapachyloides armatus* were both formerly placed in Pachylinae and transferred recently to Gonyleptinae by Kury et al. (2010). In that occasion, they justified the transfer arguing that penial features are typical of those of Gonyleptinae, but they did not formally described or characterized them. Despite the typical “Pachylinae” general aspect (four scutal areas on dorsal scutum, robust pedipalps and conspicuous trochanter armature), the penis morphology, such as the deep cleft on distal margin of the penis ventral plate, indicates that it belongs to a more derivative gonyleptine lineage.

### Heteropachylinae Kury, 1994a

#### Pseudopucrolia

Roewer,1912

http://species-id.net/wiki/Pseudopucrolia

Pseudopucrolia Roewer, 1912: 167; Mendes 2011: 451; (type species: *Pseudopucrolia spinosa* Roewer, 1912, by monotypy).
Meteusarcus Roewer, 1913: 74; Kury 2003: 177; (type species: *Meteusarcus armatus* Roewer, 1913, by monotypy). **Syn. n.**

##### Diagnosis.

See Mendes (2011: 453).

**Composition**. *Pseudopucrolia discrepans* (Roewer, 1943); *Pseudopucrolia incerta* (Mello-Leitão, 1928); *Pseudopucrolia mutica* (Perty, 1833) and *Pseudopucrolia rugosa* (Roewer, 1930).

##### Taxonomical note.

*Meteusarcus armatus* was formerly placed in Gonyleptidae, Pachylinae. The examination of the female holotype revealed it was a female of the heteropachyline *Pseudopucrolia mutica*.

#### Pseudopucrolia mutica

(Perty, 1833)

http://species-id.net/wiki/Pseudopucrolia_mutica

Eusarcus muticus Perty, 1833: 203.
Pseudopucrolia mutica : Kury 2003: 143; Mendes 2011: 462.
Meteusarcus armatus Roewer, 1913: 74, fig. 33 (♀); Kury 2003: 177; (female holotype; Brazil; SMF 801; examined). **Syn. n.**

##### Diagnosis.

See Mendes (2011: 463).

##### Taxonomical note.

See genus “Taxonomical note”.

### Pachylinae Sørensen, 1884

#### Acrographinotus

Holmgren, 1916

http://species-id.net/wiki/Acrographinotus

Acrographinotus Holmgren, 1916: 89; Roewer 1929: 240; Kury 2003: 155; (type species: *Acrographinotus erectispina* Roewer, 1929, by subsequent designation of Roewer (1929)).
Unduavius Roewer, 1929: 244; Kury 2003: 195; (type species: *Unduavius ornatus* Roewer, 1929, by monotypy). **Syn. n.**

##### Diagnosis.

See Acosta (2001: 59).

##### Composition.

*Acrographinotus ceratopygus* (Soares & Bauab-Vianna, 1972); *Acrographinotus curvispina* Roewer, 1929; *Acrographinotus erectispina* Roewer, 1929; *Acrographinotus mitmaj* Acosta, 2002; *Acrographinotus niawpaq* Acosta, 2001; *Acrographinotus ortizi* (Roewer, 1957); *Acrographinotus ornatus* (Roewer, 1929), **comb. n.**

##### Taxonomical note.

The genus *Unduavius* is monotypic, but the remarkable ventral process of the penis, with its “ibis head” shape, fits perfectly in the generic concept presented by Acosta (2001), who rediagnosed the genus *Acrographinotus*.

#### Acrographinotus ornatus

(Roewer, 1929) comb. n.

http://species-id.net/wiki/Acrographinotus_ornatus

Fig. 16

Unduavius ornatus Roewer, 1929: 244, fig. 28 (♂); Kury 2003: 195; (syntypes from NHMW, not examined, probably lost).

##### Material examined.

BOLIVIA. La Paz: Nor Yungas (Unduavi), 1 male & 6 females (SMF 2881/79; syntypes according to Acosta (1996), which was previously referred to as belonging to Naturhistorisches Museum, Wien. We acknowledge them as syntypes together with those in SMF RII 995/52).

##### Diagnosis.

*Acrographinotus ornatus* can be easily distinguished from other species of the genus by the blister-like, enlarged, brown tubercles on whitish scutal areas I–IV; posterior margin of dorsal scutum and free tergites I–II with a row of enlarged tubercles and white spots on lateral and posterior margin of dorsal scutum and coxa IV.

##### Description.

**Figure 16.**
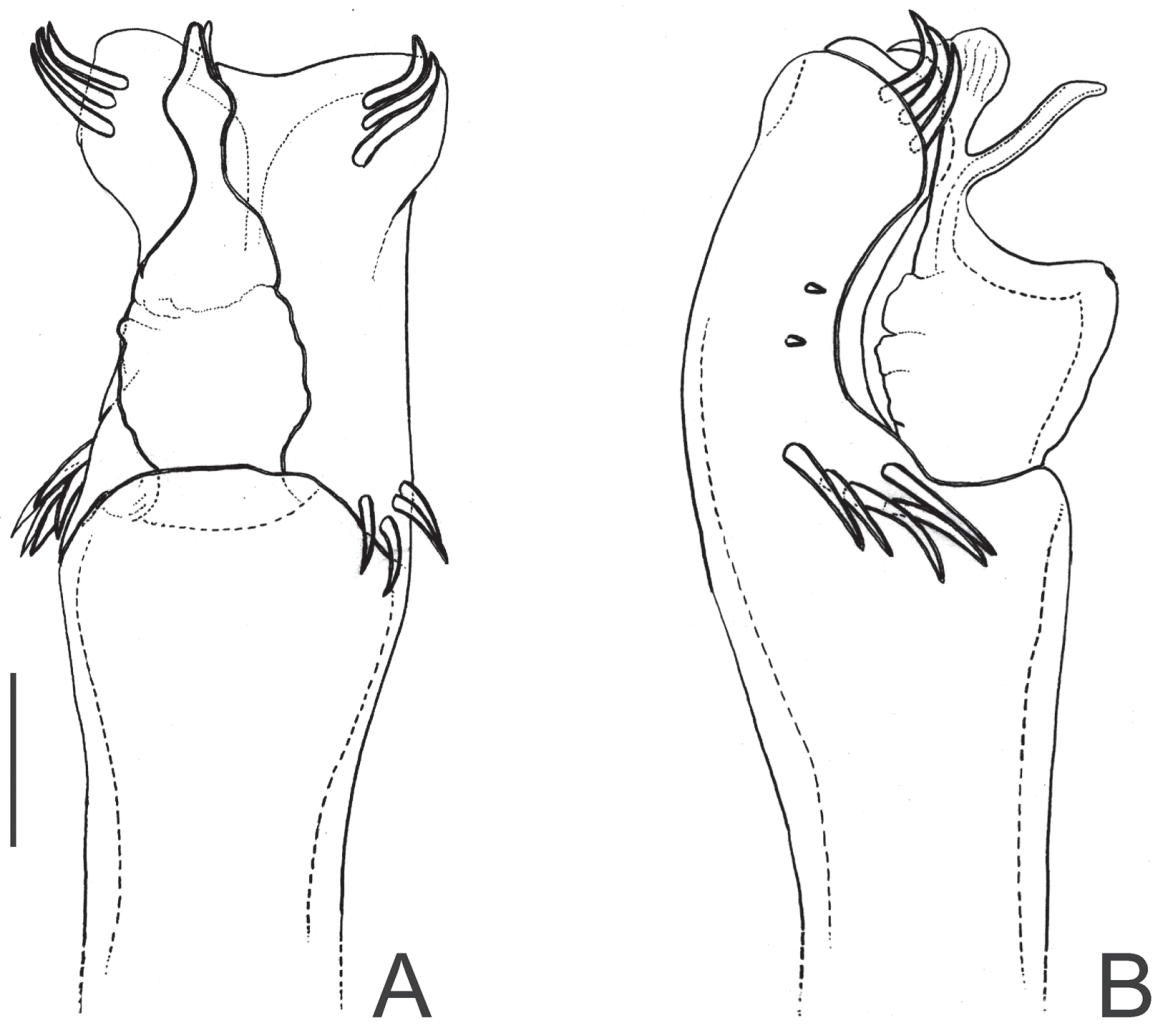
*Acrographinotus ornatus* (Roewer) **comb. n.** Distal part of penis (SMF 2881) **A** in dorsal view **B** ditto, left lateral view. Scale bar: 0.1 mm.

Penis (Fig. 16A–B; SMF 2881): ventral plate with 3 distal pairs of long setae, 0–2 median pairs of short setae, 4–5 basal pairs of setae. Stylus almost straight (apex slightly bent), smooth. Distal ventral process shaped as an ibis head.

#### Gyndesops

Roewer, 1943 new subfamilial assignment

http://species-id.net/wiki/Gyndesops

Gyndesops Roewer, 1943: 29; Kury 2003: 129; (type species: *Gyndesops denisi* Roewer, 1943).

##### Diagnosis.

*Gyndesops* resembles the largest Pachylinae genus, *Discocyrtus* Holmberg, 1878, which presents an ocularium and scutal area III with paired armature, four scutal areas, scutal area II–IV and free tergites I–III unarmed. This combination of characters also occurs in genera such as *Gyndoides* Mello-Leitão, 1927, *Lacronia* Strand, 1942, *Paraluederwaldtia* Mello-Leitão, 1927 and *Parapucrolia* Roewer, 1917. *Gyndesops* can be distinguished from *Lacronia* and *Parapucrolia* by the coloration, which is uniformly auburn (*Lacronia* has green/yellow spots and/or stripes on dorsal scutum and *Parapucrolia* has white patches on dorsal scutum). It is very difficult to distinguish *Gyndesops* from the remaining genera, because no revision was ever made including those groups, and their monophyly is doubtful. It is possible that *Gyndesops* (as well as *Gyndoides* and *Paraluederwaldtia*) is a junior synonym of *Discocyrtus*, but we refrained ourselves from such a rash nomenclatural act.

##### Composition.

Monotypic.

#### Gyndesops denisi

Roewer, 1943

http://species-id.net/wiki/Gyndesops_denisi

Fig. 17

Gyndesops denisi Roewer, 1943: 29, fig. 23 (♂); Kury 2003: 129; (male holotype, Brazil [Brasilien: Nova Teutonia], SMF 8833/117, examined).

##### Description.

**Figure 17.**
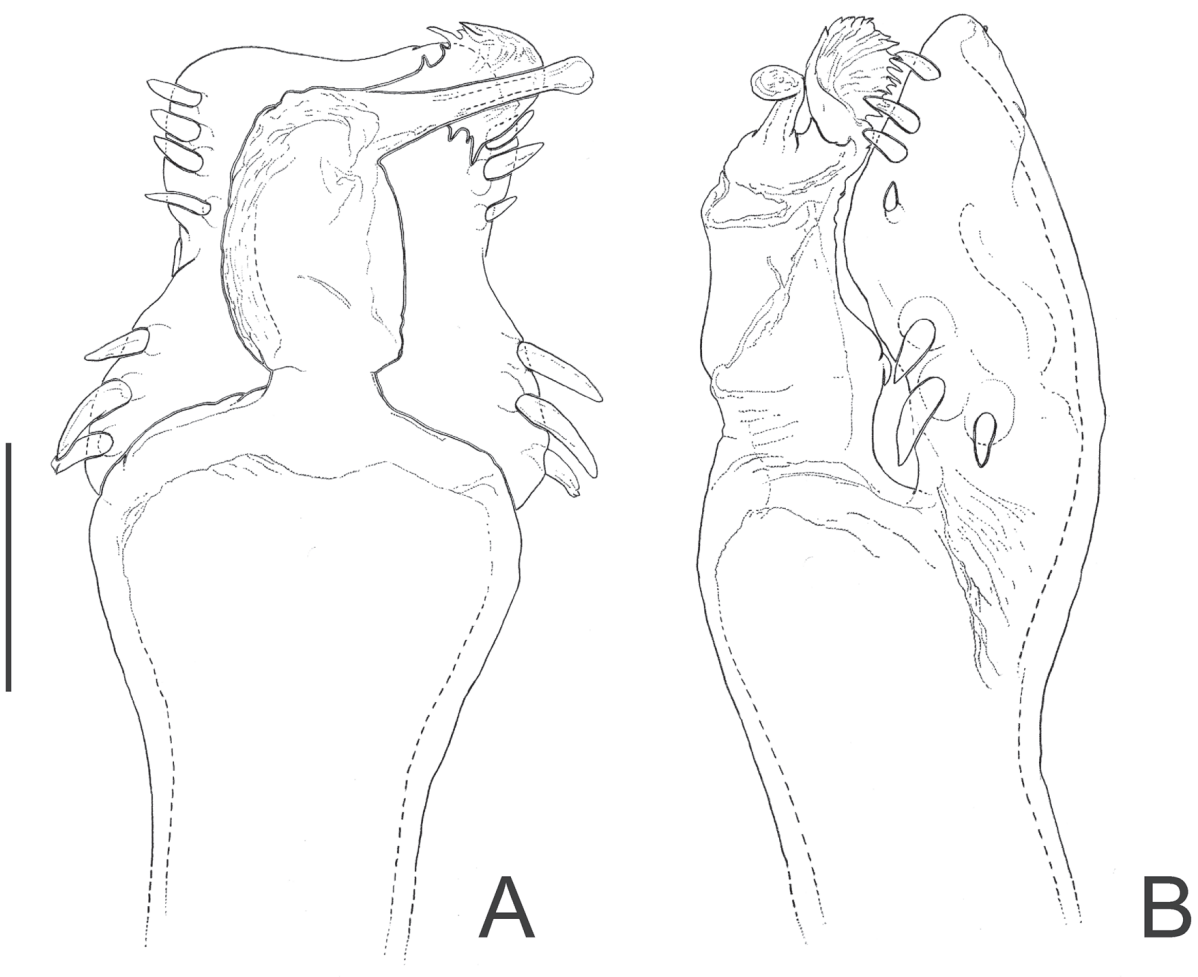
*Gyndesops denisi* Roewer. Distal part of penis (holotype) **A** in dorsal view **B** ditto, right lateral view. Scale bar: 0.1 mm.

Penis (Fig. 17A–B, holotype): ventral plate subrectangular, basal half of lateral margin projected laterad, distal margin slightly concave, 3 distal pairs of cylindrical setae, 1 pair of median setae (long left seta and moderate sized right seta), 3 basal pairs of cylindrical setae (median largest). Glans sac almost reaching distal margin of ventral plate. Stylus with swollen apex, smooth. Ventral process with large shaft, apex flabelliform with serrated lateral margins.

##### Taxonomical note.

Originally, *Gyndesops denisi* was allocated in Pachylinae, and later transferred to Gonyleptinae by Kury (2003), without further explanation. It is known that Gonyleptinae and Pachylinae are possibly not monophyletic. A phylogenetic analysis of Gonyleptidae is currently being carried out and indicates, however, that most Gonyleptinae have a deep cleft in the distal margin of the penis ventral plate, as well as some other features, absent in this species. Since *Gyndesops denisi* (i) does not possess such cleft in the penis ventral plate, and (ii) the exterior morphology might be misleading regarding Gonyleptinae and Pachylinae, we opted to allocate it back in Pachylinae.

#### Haversia

Roewer, 1913 new subfamilial assignment

http://species-id.net/wiki/Haversia

Haversia Roewer, 1913: 170; Kury 2003: 129; (type species: *Gonyleptes defensus* Butler, 1876).
Hoggellula Roewer, 1930: 397; Kury 2003: 129; (type species: *Sadocus vallentini* Hogg, 1913, by monotypy). **Syn. n.**

##### Diagnosis.

*Haversia* resembles Pachylinae with three scutal areas, such as *Corralia* Roewer, 1913, *Diconospelta* Canals, 1934, *Graphinotus* Koch, 1839, *Huassampilia* Roewer, 1913, *Neogonyleptes* Roewer, 1913, *Oxapampeus* Roewer, 1936, *Spinivunus* Roewer, 1943, *Tumbesia* Loman, 1899, *Ubatubesia* B. Soares, 1945, and two species of *Sadocus* Sørensen, 1886 (*Sadocus conspicillatus* and *Sadocus polyacanthus*). *Haversia* can be distinguished from those genera by the unarmed and smooth (i.e. without tubercles) scutal areas and the complex apophyses of male trochanter IV: three prolateral apophyses (anterior median, posterior median and an apical apophysis).

##### Taxonomical note.

The two type species (*Gonyleptes defensus* and *Sadocus vallentini*)are synonymous, resulting in a generic synonymy, and the new assignment proposed here is based on penis morphology (see note on *Gonyleptes denisi*). The penis of *Haversia* follows the basic pattern of those gonyleptids placed in the large (and non-monophyletic) subfamily Pachylinae (ventral plate with straight anterior margin; glans without dorsal process; distal, median and basal setae of ventral plate short [seta length inferior to ventral plate width]; stylus and ventral process with variable shape). However, *Haversia* presents two features that stand out: (i) the penis glans projected dorsally and (ii) the detailed shape of apex of penis ventral process. Those features are typical of some Chilean genera, such as *Fonckia*, *Neogonyleptes* and *Sadocus* (unpublished data), and might be the evidence that those four genera are closely related.

##### Composition.

Monotypic.

#### Haversia defensa

(Butler, 1876)

http://species-id.net/wiki/Haversia_defensa

Fig. 18

Gonyleptes defensus Butler, 1876: 152, fig. 4 (♂); (4 males & 1 female syntypes; Argentina, Falklands Island; NHM; examined by detailed photos).
Haversia defensa : Roewer, 1913: 171, fig. 72 (♂); Kury 2003: 129.
Sadocus vallentini Hogg, 1913: 48, fig. 7 (♂); (syntypes males and females; Falklands Island; NHM 1299.1304; not examined). **Syn. n.**
Hoggellula vallentini : Roewer 1930: 397, fig. 22 (♂); Kury 2003: 129.

##### Material examined.

ARGENTINA. Falklands (Islas Malvinas), 1 ♂ (SMF 1320/8).

##### Description.

**Figure 18.**
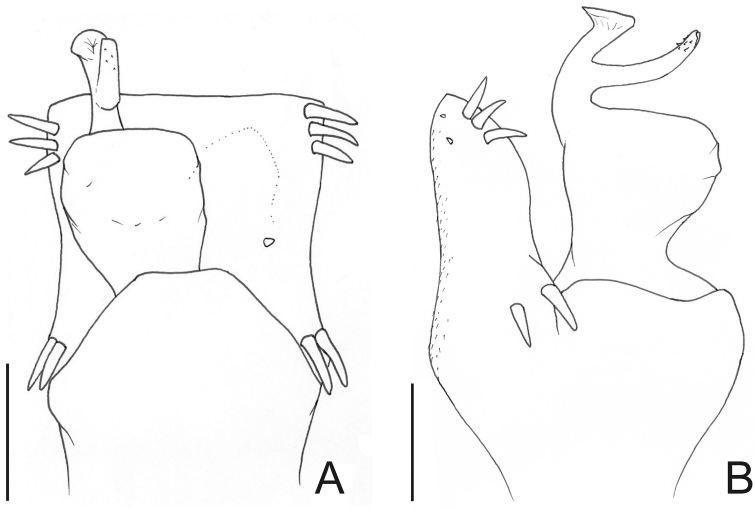
*Haversia defensa* (Butler). Distal part of penis (SMF 1320/8) **A** in dorsal view **B** ditto, left lateral view. Scale bars: 0.1 mm.

Penis (Fig. 18A–B; SMF 1320/8): ventral plate with lateral margins slightly concave, distal margin straight, 3 distal pairs of cylindrical, straight, long setae, 1 pair of median short setae, 2 pairs of basal cylindrical, straight, long setae. Glans sac projected dorsally, ventral process and stylus arising ventrally. Stylus long, slightly curved, with apical ventral and lateral trichomes. Ventral process swollen apically, with nailhead-like apex.

##### Taxonomical note.

The examination of detailed photos of *Gonyleptes defensus* type material and subsequent comparison of those with the description of *Sadocus vallentini* allowed recognizing them as conspecific.

#### Oxapampeus

Roewer,1963 new subfamilial assignment

http://species-id.net/wiki/Oxapampeus

Oxapampeus Roewer, 1963: 62; Kury 2003: 135; (type species: *Oxapampeus weyrauchi* Roewer, 1963).

##### Diagnosis.

Based on external morphology, *Oxapampeus* resembles Pachylinae with three scutal areas (subfamily classical diagnosis is the presence of four areas) and scutal area III with a paramedian pair of spines, such as *Corralia* Roewer, 1913, *Diconospelta* Canals, 1934, *Haversia* Roewer, 1913, *Huassampilia* Roewer, 1913, *Neogonyleptes* Roewer, 1913, *Spinivunus* Roewer, 1943, and two species of *Sadocus* Sørensen, 1886 (*Sadocus conspicillatus* and *Sadocus polyacanthus*). However, *Oxapampeus* can be distinguished from those genera by the combination of the following characters: ocularium with a paramedian pair of pointed tubercles; frontal hump on anterior border of dorsal scutum moderately developed; scutal areas I–II each with a paramedian pair of enlarged tubercles; posterior margin of dorsal scutum straight; and free tergites I–III unarmed.

##### Taxonomical note.

*Oxapampeus* has been included in Gonyleptinae due to the presence of three scutal areas on the dorsal scutum and absence of autapomorphies of other gonyleptidean subfamilies. However, its penis does not present the typical gonyleptine pattern. In addition, its ventral plate, with slightly concave lateral sides, as well as the absence of dorsally projected basal lobes, makes it resemble several Andean Pachylinae male genitalia.

##### Composition.

Monotypic.

#### Oxapampeus weyrauchi

Roewer, 1963

http://species-id.net/wiki/Oxapampeus_weyrauchi

Fig. 19

Oxapampeus weyrauchi Roewer, 1963: 62, fig. 29 (♂); Kury 2003: 135; (male holotype, 2 males & 5 females paratypes, Peru, Pasco, Western slope of Eastern Andes, Villarica near Oxapampa, 1500 m, in Ucayali drainage, SMF RII 13964/74, examined).

##### Description. 

**Figure 19.**
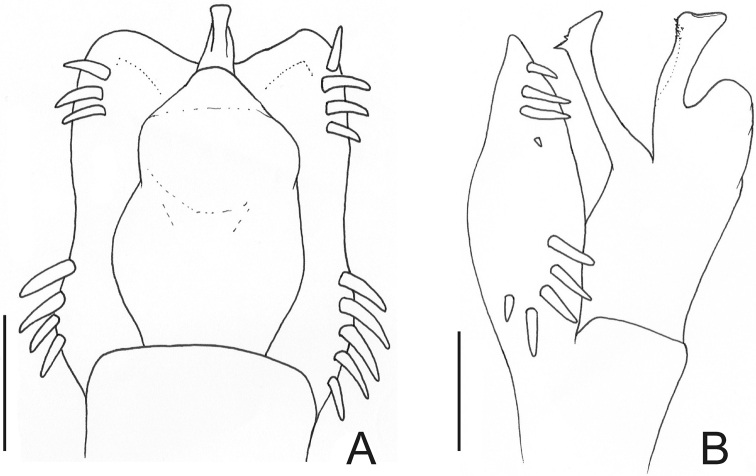
*Oxapampeus weyrauchi* Roewer. Distal part of penis (holotype) **A** in dorsal view **B** ditto, left lateral view. Scale bars: 0.1 mm.

Penis (Fig. 19A–B; holotype): ventral plate with lateral and distal margins slightly concave, with 3–4 distal pairs of cylindrical, straight, long setae, 0–1 median seta (visible in lateral view), 5 basal pairs of setae. Glans sac projected dorsally, ventral process and stylus arising ventrally. Stylus short and straight, with ventral subapical trichomes. Ventral process swollen apically, more elongated and thinner than stylus.

## Supplementary Material

XML Treatment for Nemastygnus

XML Treatment for Nemastygnus ovalis

XML Treatment for Taulisa

XML Treatment for Taulisa koepckei

XML Treatment for Napostygnus

XML Treatment for Napostygnus bispinosus

XML Treatment for Neopachyloides peruvianus

XML Treatment for Pirunipygus

XML Treatment for Pirunipygus paradoxus

XML Treatment for Acanthogonyleptes

XML Treatment for Acanthogonyleptes singularis

XML Treatment for Geraeocormobius sylvarum

XML Treatment for Gonyleptellus bimaculatus

XML Treatment for Gonyleptes

XML Treatment for Gonyleptes horridus

XML Treatment for Gonyleptes curvicornis

XML Treatment for Gonyleptes atrus

XML Treatment for Gonyleptes fragilis

XML Treatment for Gonyleptes perlatus

XML Treatment for Gonyleptes pustulatus

XML Treatment for Liogonyleptoides minensis

XML Treatment for Mischonyx

XML Treatment for Mischonyx scaber

XML Treatment for Mischonyx cuspidatus

XML Treatment for Megapachylus grandis

XML Treatment for Parampheres

XML Treatment for Parampheres tibialis

XML Treatment for Parapachyloides uncinatus

XML Treatment for Schubartesia singularis

XML Treatment for Pseudopucrolia

XML Treatment for Pseudopucrolia mutica

XML Treatment for Acrographinotus

XML Treatment for Acrographinotus ornatus

XML Treatment for Gyndesops

XML Treatment for Gyndesops denisi

XML Treatment for Haversia

XML Treatment for Haversia defensa

XML Treatment for Oxapampeus

XML Treatment for Oxapampeus weyrauchi
